# Targeting Itga8 Mitigates Neurogenic Bladder Fibrosis Driven by Trem2⁺ Macrophage‐Derived Fn1 via FAK/RhoA/ROCK Signaling

**DOI:** 10.1002/advs.202510631

**Published:** 2025-12-08

**Authors:** Jiaxin Wang, Siyuan Wang, Lida Ren, Xinqi Liu, Lei Zhang, Peng Hu, Wenchao Xu, Shuaixiang Zheng, Jihong Liu, Qing Ling

**Affiliations:** ^1^ Department of Urology Tongji Hospital Tongji Medical College Huazhong University of Science and Technology Wuhan 430030 China; ^2^ Institute of Urology Tongji Hospital Tongji Medical College Huazhong University of Science and Technology Wuhan 430030 China; ^3^ Department of Geriatrics Tongji Hospital Tongji Medical College Huazhong University of Science and Technology Wuhan 430030 China; ^4^ Wuhan Silicon‐life Medical Technology Co., LTD Wuhan 430030 China

**Keywords:** extracellular matrix remodeling, fibrosis, Itga8^+^ fibroblasts, neurogenic bladder, Trem2^+^ macrophages

## Abstract

Neurogenic bladder (NB)‐induced fibrosis is the major cause of irreversible bladder dysfunction, yet the underlying mechanisms remain undefined. Here, leveraging single‐cell RNA sequencing, the fibrotic landscape of NB is delineated and a distinct integrin α8 (Itga8) ⁺ fibroblast population. The Itga8⁺ fibroblasts expand substantially during the acute phase post‐injury and exhibit a fibrogenic transcriptional profile. Mechanistically, Itga8 is found to coordinate cytoskeletal remodeling via the FAK/RhoA/ROCK signaling to facilitate fibroblast activation. Moreover, fibroblast activation is orchestrated by Trem2⁺ macrophages, which secrete Fn1 to engage Itga8 on fibroblasts, thereby reinforcing the pro‐fibrotic communication between fibroblasts and macrophages. Notably, macrophage depletion markedly attenuates fibrosis and restores bladder function, underscoring their pivotal role in NB pathogenesis. In vivo, conditional deletion of *Itga8* (*Col1a2*‐CreERT; *Itga8* ^fl/fl^) or local knockdown of *Itga8* significantly attenuates collagen deposition and improves voiding efficiency. Collectively, this study reveals a novel Itga8‐centered fibroinflammatory axis and nominates Itga8 as a promising therapeutic target for delaying fibrosis progression and restoring bladder function.

## Graphical abstract

Normal bladders exhibit quiescent fibroblasts/macrophages, whereas neurogenic bladders show acute‐phase Itga8⁺ fibroblast expansion driven by Trem2⁺ macrophage‐secreted Fn1, which activates FAK/RhoA/ROCK signaling, promotes cytoskeletal remodeling, and upregulates pro‐fibrotic genes. Itga8 inhibition blocks these pathways, delays fibrogenesis, and preserves bladder function.

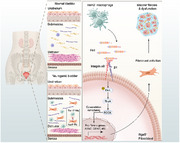

## 1 Introduction

Neurogenic bladder (NB) is a debilitating condition resulting from damage to the central or peripheral nervous system, characterized by impaired bladder storage and voiding, which leads to deteriorating renal function and a significant impact on the patient's quality of life.^[^ ^1^, ^2^, ^3^ ^]^ As the disease progresses, the majority of NB patients develop varying degrees of bladder fibrosis, which leads to irreversible deterioration of bladder function.^[^ ^4^, ^5^ ^]^ Current therapeutic approaches, such as intermittent catheterization and anticholinergic medications, provide temporary relief from lower urinary tract symptoms but fail to address the underlying pathophysiological mechanisms of fibrosis.^[^ ^6^ ^]^ Although delaying fibrosis progression has been recognized as a critical objective by the International Continence Society (ICS), there remains an unmet need for targeted therapies that specifically address NB‐induced fibrosis.^[^ ^7^, ^8^ ^]^

Fibrosis is characterized by the excessive accumulation and deposition of the extracellular matrix (ECM) within tissues, primarily driven by the activation of fibroblasts.^[^ ^9^ ^]^ Within the bladder, this fibrotic remodeling contributes to the disruption of normal urodynamics, manifesting as decreased bladder compliance, increased residual urine volume, and diminished voiding efficiency.^[^ ^10^ ^]^ Despite the well‐established role of fibroblasts in ECM production, the cellular and molecular mechanisms underlying fibroblast activation in the context of NB remain poorly understood. Recent advances in single‐cell transcriptomics have revealed the intricate heterogeneity of fibroblast populations in fibrotic tissues, uncovering novel subtypes with distinct roles in disease progression.^[^ ^11^, ^12^, ^13^ ^]^ However, the specific fibroblast subsets contributing to neurogenic fibrosis have yet to be fully defined in the bladder. It is therefore imperative to elucidate the key fibroblast subpopulations involved in mediating NB fibrosis, as well as the molecular mechanisms driving their activation, in order to develop effective therapeutic strategies.

While fibroblasts have been considered as the primary drivers of ECM deposition, emerging evidence underscores the critical role of immune cell‐derived signals in orchestrating fibroblast activation and phenotypic transition.^[^ ^14^ ^]^ In response to tissue injury, immune cells, especially macrophages, critically regulate fibroblast activity and thereby govern the progression of fibrotic remodeling.^[^ ^15^ ^]^ In recent years, single‐cell RNA sequencing (scRNA‐seq) has revealed that certain macrophage subpopulations, such as SPP1⁺,^[^ ^16^ ^]^ CCR^hi^,^[^ ^17^ ^]^ and CD38^hi[^ ^18^ ^]^ macrophages, activate fibroblasts through paracrine signaling, which challenges the traditional M1 (pro‐inflammatory) / M2 (pro‐fibrotic) polarization model.^[^ ^19^ ^]^ In the context of NB, nerve injury triggers the acute‐phase infiltration of macrophages and the release of inflammatory cytokines (e.g., IL‐1β, IL‐6, TNF‐α), which collectively contribute to the progression of fibrosis.^[^ ^20^ ^]^ Although immune‐fibroblast crosstalk is a critical component of fibrogenesis, the specific macrophage subset involved and the exact molecular dialogue remain poorly understood, highlighting the pressing need for further investigation into the immune‐fibroblast axis in neurogenic fibrosis.

Here, with scRNA‐seq, we identified a distinctive Itga8⁺ fibroblast subpopulation that expands rapidly after nerve injury and exhibits a fibrogenic transcriptional program characterized by cytoskeleton remodeling and ECM production. Mechanistically, Itga8 promoted fibroblast activation through FAK/RhoA/ROCK signaling, and engaged with Trem2⁺ macrophage‐derived fibronectin (Fn1) to form a potent pro‐fibrotic signaling axis. In vivo, lentivirus‐mediated localized knockdown of Itga8 in the bladder attenuated fibrotic progression and preserved bladder function. Similarly, in a fibroblast‐specific knockout mouse model (*Col1a2*‐CreERT; *Itga8* ^fl/fl^), deletion of *Itga8* markedly reduced fibrotic remodeling and maintained bladder functional integrity. Collectively, our findings identified Itga8⁺ fibroblasts as central contributors to NB fibrosis and suggested that targeting Itga8 represented a promising therapeutic strategy for halting fibrosis progression and restoring bladder function in neurogenic disorders.

## 2 Results

### 2.1 Early Fibroblast Activation Drives Denervation‐Induced Bladder Fibrosis

**Figure 1:**
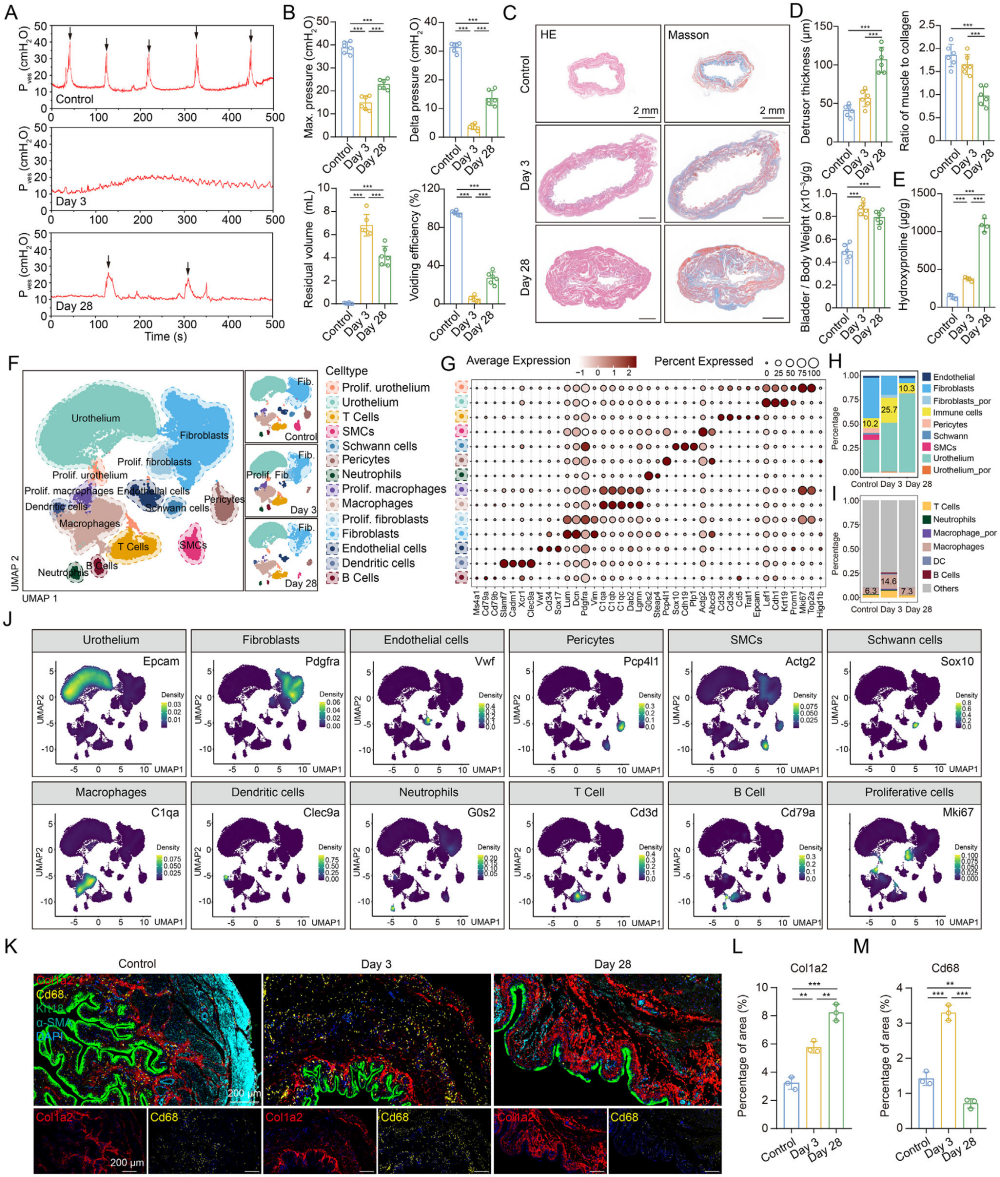
Single‐cell sequencing reveals the dynamic changes in the microenvironment during the acute and chronic phases of neurogenic bladder fibrosis. A) Representative urodynamic features of the NB rat model, and the black arrow indicates a single voiding event. B) Quantification of maximum intravesical pressure, delta intravesical pressure, residual volume, and voiding efficiency (voided volume/maximum bladder capacity) in (A), n = 6 per group. C) Representative HE and Masson's trichrome staining of bladder tissues. D) Quantification of detrusor thickness, ratio of smooth muscle to collagen, and bladder to body weight ratio in (C), n = 6 per group. E) Hydroxyproline content of control bladder or NB, n = 4 per group. F) Uniform manifold approximation and projection (UMAP) plots showing 14 major cell types from control (n = 3), day 3 (n = 3), and day 28 (n = 2) groups. G) Dot plots of representative markers in the indicated major cell types. The average gene expression and percentage of cells expressed are shown by dot color and size, respectively. H) Proportion of total immune cells in each group. I) Proportion of various immune cell types in each group. J) UMAP showing the expression of marker genes of major cell types. K) Representative immunofluorescence images of Krt18 (green), Col1a2 (red), Cd68 (yellow), α‐SMA (cyan), and DAPI (blue) expression in different groups. L) Quantitative analysis of Col1a2 in immunofluorescence staining, n = 3 per group. M) Quantitative analysis of Cd68 in immunofluorescence staining, n = 3 per group. P_ves_ = intravesical pressure; Max. = maximum; Prolif. = Proliferative; Fib. = Fibroblasts. Data represent mean ± SD. One‐way ANOVA was used for B, D, E, L, and M. ^**^ *p* < 0.01, ^***^ *p* < 0.001.

To investigate the contribution of denervation to bladder remodeling, we performed urodynamics assessment and histological analysis in rats subjected to bilateral pelvic nerve injury (BPNI) at 3 days (acute phase) and 28 days (chronic phase) post‑injury (Figure S1A, Supporting Information). In the acute phase, maximal intravesical pressure, delta voiding pressure, and voiding efficiency were markedly reduced, while post‐void residual volume was significantly increased. These parameters exhibited only partial recovery during the chronic phase (**Figure** **1**A,B). Ex vivo detrusor contraction tests demonstrated a transition from early hypercontractility to subsequent dysfunction (Figure S1B–D, Supporting Information). Hematoxylin and Eosin (HE) and Masson's trichrome staining revealed detrusor muscle thickening, an increased bladder‑to‑body‐weight ratio, and a reduced muscle‑to‑collagen ratio (Figure 1C,D). Moreover, hydroxyproline, a key amino acid component of collagen, progressively increased following denervation (Figure 1E). Together, these results indicated that the BPNI model recapitulated both the functional deterioration and fibrotic remodeling characteristic of NB.

Next, we systematically mapped denervation‐induced remodeling of the bladder microenvironment by performing scRNA‐seq on 56740 high‐quality cells isolated from sham‐operated rats and from BPNI rats at 3‐ and 28‐days post‐injury (Figure 1F). Uniform manifold approximation and projection (UMAP) dimensionality reduction and clustering delineated 14 distinct cell populations, including urothelial cells (*Epcam*, *Cdh1*, *Krt19*), fibroblasts (*Lum*, *Dcn*, *Vim*, *Pdgfra*), smooth muscle cells (*Actg2*), pericytes (*Pcp4l1*), macrophages (*C1qa*, *C1qb*, *C1qc*, *Dab2*, *Lgmn*), neutrophils (*G0s2*, *Steap4*), T cells (*Cd3d*, *Cd3e*), B cells (*Ms4a1*, *Cd79a*, *Cd79b*), Schwann cells (*Sox10*, *Cdh19*, *Plp1*), and proliferative subsets of urothelial cells, fibroblasts, and macrophages (co‐expressing lineage markers along with *Mki67* and *Top2a*) (Figure 1G,J). Functional enrichment analysis supported the biological identities of these clusters, further validating the classification (Figure S2A, Supporting Information). Notably, the acute phase was marked by a pronounced expansion of proliferative fibroblasts and an increase in fibroblast heterogeneity, underscoring fibroblasts as a principal cellular group affected by denervation (Figure 1F). Consistent with these observations, bulk RNA‐seq of bladders from acutely and chronically denervated models revealed early activation of fibrosis‐promoting pathways, including TGF‑β/Smad (NES =  1.58, *p* < 0.05) and PI3K‑Akt (NES =  1.30, *p* < 0.05) signaling pathway (Figure S2B–F, Supporting Information). In parallel, substantial increases in the proportions of macrophages, T cells, and B cells were observed, suggesting a potential role for immune‐stromal crosstalk in the early response to nerve injury (Figure 1H,I). The dynamic expansion of fibroblasts and macrophages during the acute phase was further confirmed by immunofluorescence, supporting the transcriptomic findings (Figure 1K–M). Collectively, these data demonstrated that fibroblast activation and pro‑fibrotic signaling are rapidly initiated following denervation, establishing the foundation for subsequent ECM accumulation and fibrotic remodeling.

### 2.2 Itga8⁺ Fibroblasts Represent a Pro‐Fibrotic Subpopulation Driving Bladder Fibrosis After Nerve Injury

**Figure 2:**
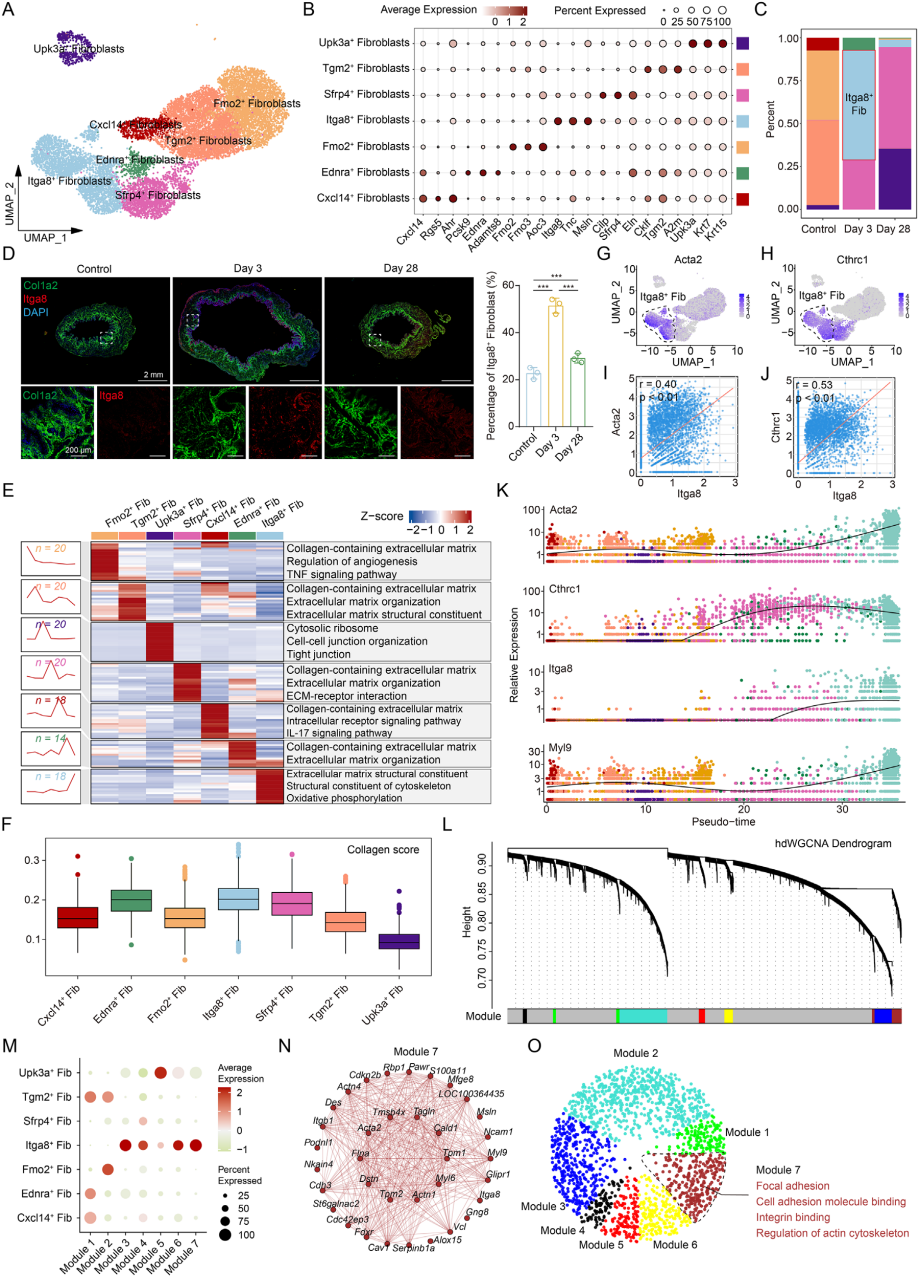
Itga8^+^ fibroblasts represent a critical subpopulation driving neurogenic bladder fibrosis. A) UMAP plot of subclustered fibroblasts. B) Dot plot of markers for each subset. C) Frequency of each cluster at different time points. D) Representative immunofluorescence images and quantitative analysis of Col1a2 (green), Itga8 (red), and DAPI (blue) expression in different groups, n = 3 per group. E) Representative Gene Ontology (GO) and Kyoto Encyclopedia of Genes and Genomes (KEGG) enrichment of the marker genes expressed in each subset of fibroblasts. F) Box plots of the collagen signature score for each fibroblast subset. G) UMAP plot of *Acta2*. H) UMAP plot of *Cthrc1*. I,J) Scatter plot demonstrating a significant positive correlation between *Itga8* and (I) *Acta2* or J) *Cthrc1*. K) Dot plots demonstrated the expression dynamics of *Acta2*, *Cthrc1*, *Itga8*, and *Myl9* along pseudo‐time. L) The hdWGCNA dendrogram of seven identified modules. M) A bubble plot represented the scores of the seven modules in each subset. N) Networks of the representative genes from module 7. O) Enrichment analysis of each module. Fib. = Fibroblasts. Data represent mean ± SD. One‐way ANOVA was used for D. ^***^ *p* < 0.001.

To identify the fibroblast subpopulation driving bladder fibrosis following nerve injury, we classified fibroblasts into seven transcriptionally distinct subclusters based on signature gene expression profiles (**Figure** **2**A,B). Interestingly, we identified a subset of fibroblasts that express the urothelial marker gene *Upk3a*. Pseudotime analysis and immunofluorescence staining suggested that these cells might originate from urothelial cells, indicating the possible occurrence of epithelial‐mesenchymal transition (EMT) during the progression of NB (Figure S3, Supporting Information). Among the fibroblast subpopulation, Itga8^+^ fibroblasts markedly accumulated during the acute phase of BPNI (Figure 2C; Figure S4A,B, Supporting Information), as confirmed by immunofluorescence (Figure 2D). This subset was enriched for gene programs related to ECM structural constituents, cytoskeletal components, and oxidative phosphorylation, suggesting active cytoskeletal remodeling and ECM production (Figure 2E). Correspondingly, Itga8⁺ fibroblasts exhibited the highest collagen gene signature score among all fibroblast subclusters, reflecting their dominant collagen‐producing potential (Figure 2F). Moreover, Itga8⁺ fibroblasts showed elevated expression of *Acta2* and *Cthrc1*, key markers of fibroblast activation and fibrogenesis, compared to other subsets (Figure 2G,H). Correlation analysis revealed significant positive associations between *Itga8* and both *Acta2* (Pearson r = 0.40, *p* < 0.01) and *Cthrc1* (Pearson r = 0.53, *p* < 0.01), indicating coordinated upregulation of fibrosis‐inducing genes within this population (Figure 2I,J). Notably, the absence of *Actg2* and *Myh11* expression in Itga8⁺ fibroblasts underscored their differential transcriptional identity compared to canonical SMCs (Figure S4C,D, Supporting Information). In addition, pseudotime trajectory analysis indicated elevated expression of fibrosis‐related genes, including *Myl9*, *Igfbp4*, *Mmp14*, *Tgfbi*, and *Thbs2*, in Itga8⁺ fibroblasts also exhibited (Figure 2K; Figure S4E,F, Supporting Information). Module‐level trajectory analysis further revealed enrichment in pathways regulating cell adhesion, regulation of actin filament length, and fibroblast activation (Figure S4G–J, Supporting Information). In contrast to the Sfrp4⁺ fibroblasts, which also increase after nerve injury, Itga8⁺ fibroblasts emerge at an earlier stage and exhibit more pronounced characteristics in contractility, ECM production, and inflammation response (Figure S5, Supporting Information). To dissect co‐expression programs, we applied high‐dimensional weighted gene co‐expression network analysis (hdWGCNA)^[^ ^21^ ^]^ and identified seven gene modules within fibroblasts after selecting the optimal soft‐power threshold (n = 4) (Figure 2L; Figure S6A, Supporting Information). Modules 7 and 3 correlated strongly and were predominantly enriched in the Itga8⁺ fibroblast subpopulation (Figure 2M; Figure S6B,C, Supporting Information). Notably, module 7, which contained Itga8, exhibited strong co‐expression with fibrosis‐associated genes such as *Tagln*, *Myl6*, and *Tpm1* (Figure 2N). Enrichment analysis highlighted pathways involved in focal adhesion, cell adhesion molecule binding, integrin binding, and regulation of the actin cytoskeleton, suggesting a functional program consistent with fibrotic activation (Figure 2O; Figure S6D,E, Supporting Information). Together, these findings established Itga8⁺ fibroblasts as a rapidly expanding, transcriptionally activated population that likely serves as a key effector of denervation‐induced bladder fibrosis.

### 2.3 Itga8 Drives Fibroblast Activation via FAK/RhoA/ROCK Signaling Pathway

**Figure 3:**
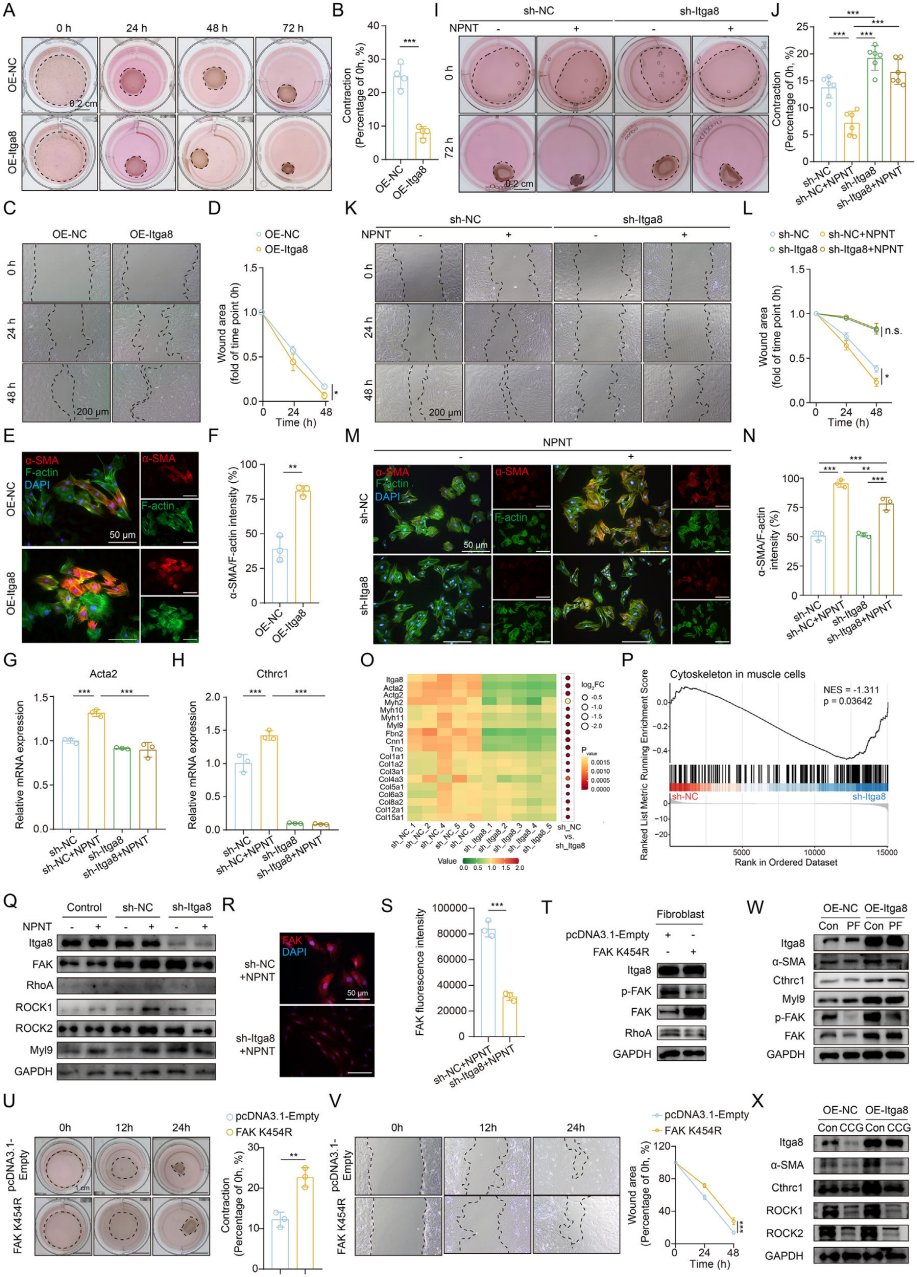
Itga8 promotes fibroblast contraction and migration via the FAK and RhoA/ROCK signaling pathways. A) Representative images of the contraction assay in fibroblasts transduced with OE‐NC or OE‐Itga8 lentivirus. B) Quantification of cell contraction in (A), n = 4 per group. C) Representative images of the scratch assay in fibroblasts transduced with OE‐NC or OE‐Itga8 lentivirus. D) Statistical analysis of the wound area over time in (C), n = 3 per group. E) Representative images of immunofluorescence staining in fibroblasts transduced with OE‐NC or OE‐Itga8 lentivirus. F) Quantification of α‐SMA/F‐actin in (E), n = 3 per group. G) qRT‐PCR analysis of *Acta2* mRNA expression, n = 3 per group. H) qRT‐PCR analysis of *Cthrc1* mRNA expression, n = 3 per group. I) Representative images of the contraction assay in fibroblasts transduced with sh‐NC or sh‐Itga8 lentivirus. J) Quantification of cell contraction in (I), n = 6 per group. K) Representative images of the scratch assay in fibroblasts transduced with sh‐NC or sh‐Itga8 lentivirus. L) Statistical analysis of the wound area over time in (K), n = 3 per group. M) Representative images of immunofluorescence staining in fibroblasts transduced with sh‐NC or sh‐Itga8 lentivirus. N) Quantification of α‐SMA/F‐actin in (M), n = 3 per group. O) RNA sequencing of primary bladder fibroblasts transduced with sh‐Itga8 or sh‐NC. The heatmap shows the downregulation of fibroblast activation‐related genes in sh‐Itga8 transduced cells. P) Gene set enrichment analysis (GSEA) revealed downregulation of the pathway regulating the actin cytoskeleton in the sh‐Itga8 group. Q) Representative images of Western blots of Itga8 and downstream molecules. R) Representative immunofluorescence images of FAK. S) Quantification of FAK fluorescence intensity in (R). T) Western blot analysis of phosphorylated FAK (p‐FAK), total FAK, and RhoA in primary bladder fibroblasts transfected with either a kinase‐dead FAK mutant (K454R). U) Representative images and quantification of the contraction assay in fibroblasts transduced with FAK K454R mutant plasmid. V) Representative images and quantification of the scratch assay in fibroblasts transduced with FAK K454R mutant plasmid. W,X) Representative Western blot images of fibroblasts overexpressing Itga8 treated with the (W) FAK inhibitor PF‐573228 or the (X) Rho kinase inhibitor CCG‐1423. n.s. = not statistically significant; NPNT = nephronectin; PF = PF‐573228; CCG = CCG‐1423. Data represent mean ± SD. Unpaired two‐tailed t‐test was used for B, F, S, and U. One‐way ANOVA was used for G, H, J, and N. Two‐way ANOVA was used for D, L, and V. ^*^ *p* < 0.05, ^**^ *p* < 0.01, ^***^ *p* < 0.001.

As a member of the integrin receptor family, Itga8 functions as a pivotal transducer of biochemical and mechanical signals between fibroblasts and the ECM, thereby modulating cytoskeletal architecture and ECM dynamics.^[^ ^22^ ^]^ To elucidate its role in fibroblast activation, we isolated primary bladder fibroblasts and confirmed their identity by immunofluorescence (Figure S7A,B, Supporting Information). Lentiviral overexpression of Itga8 led to a significant increase in the expression of α‐SMA and Cthrc1 (Figure S7C–E, Supporting Information). Functionally, Itga8 overexpression enhanced fibroblast contractility (**Figure** **3**A,B) and migratory capacity (Figure 3C,D), accompanied by an increased α‐SMA/F‐actin ratio, indicative of cytoskeletal remodeling (Figure 3E,F). To further delineate the function of Itga8, we silenced Itga8 using shRNA‐expressing lentiviruses (Figure S7F–H, Supporting Information) and generated recombinant nephronectin (NPNT), a specific ligand that selectively activates Itga8 signaling without affecting other integrins^[^ ^23^ ^]^ (Figure S8, Supporting Information). Itga8 knockdown suppressed *Acta2* and *Cthrc1* expression and blunted NPNT‐mediated fibroblast activation (Figure 3G,H). NPNT‐driven fibroblast contraction (Figure 3I,J) and migration (Figure 3K,L) were also markedly reduced upon Itga8 silencing. Immunofluorescence revealed that NPNT‐induced increase in α‐SMA/F‐actin ratios was abolished by Itga8 depletion (Figure 3M,N). Thus, Itga8 is a pivotal mediator of fibroblast activation, required for fibrotic structural remodeling.

To elucidate the downstream mechanisms involved in fibroblast activation, RNA‐seq was performed on primary bladder fibroblasts with or without Itga8 knockdown (Figure S9A–D, Supporting Information). Canonical activation markers (*Acta2*), ECM genes (*Col1a1*, *Col1a2*, *Col3a1* et al.), pro‐fibrotic regulators (*Cnn1*, *Tnc*) and cytoskeletal protein (*Myh10*, *Myh11, Myl9*) were decreased upon *Itga8* silencing (Figure 3O). Gene Set Enrichment Analysis (GSEA) revealed significant suppression of pathways related to the cytoskeleton in muscle cells and supramolecular fiber organization in the *Itga8*‐knockdown group (Figure 3P; Figure S9E,F, Supporting Information). Collectively, these transcriptional alterations indicate that *Itga8* depletion impairs fibroblast activation by disrupting cytoskeletal organization, leading us to investigate the FAK‐RhoA‐ROCK pathway—a plausible downstream mechanism for integrin‐mediated mechanosensing and cytoskeletal remodeling. NPNT‐induced upregulation of FAK, RhoA, ROCK1/2, and Myl9 was blunted by Itga8 silencing (Figure 3Q). Immunofluorescence further confirmed reduced FAK expression in Itga8‐silenced cells (Figure 3R,S). To confirm the functional linkage between Itga8 and the FAK/RhoA axis, we transfected a kinase‐dead FAK mutant (K454R) into the primary bladder fibroblasts (Figure 3T). This intervention effectively suppressed FAK phosphorylation and RhoA activity, and consequently attenuated fibroblast contractility and migration (Figure 3U,V). Furthermore, pharmacological inhibition of FAK (PF‐573228) or Rho kinase (CCG‐1423) abrogated the pro‐activating effects of Itga8 overexpression, validating these pathways as critical mediators of Itga8‐dependent fibroblast activation (Figure 3W,X). These results demonstrated that FAK/RhoA/ROCK signaling was a key downstream effector mediating Itga8‐driven fibroblast activation.

### 2.4 Trem2⁺ Macrophages Orchestrate Fibroblast Activation via Fn1‐Itga8 Signaling

**Figure 4:**
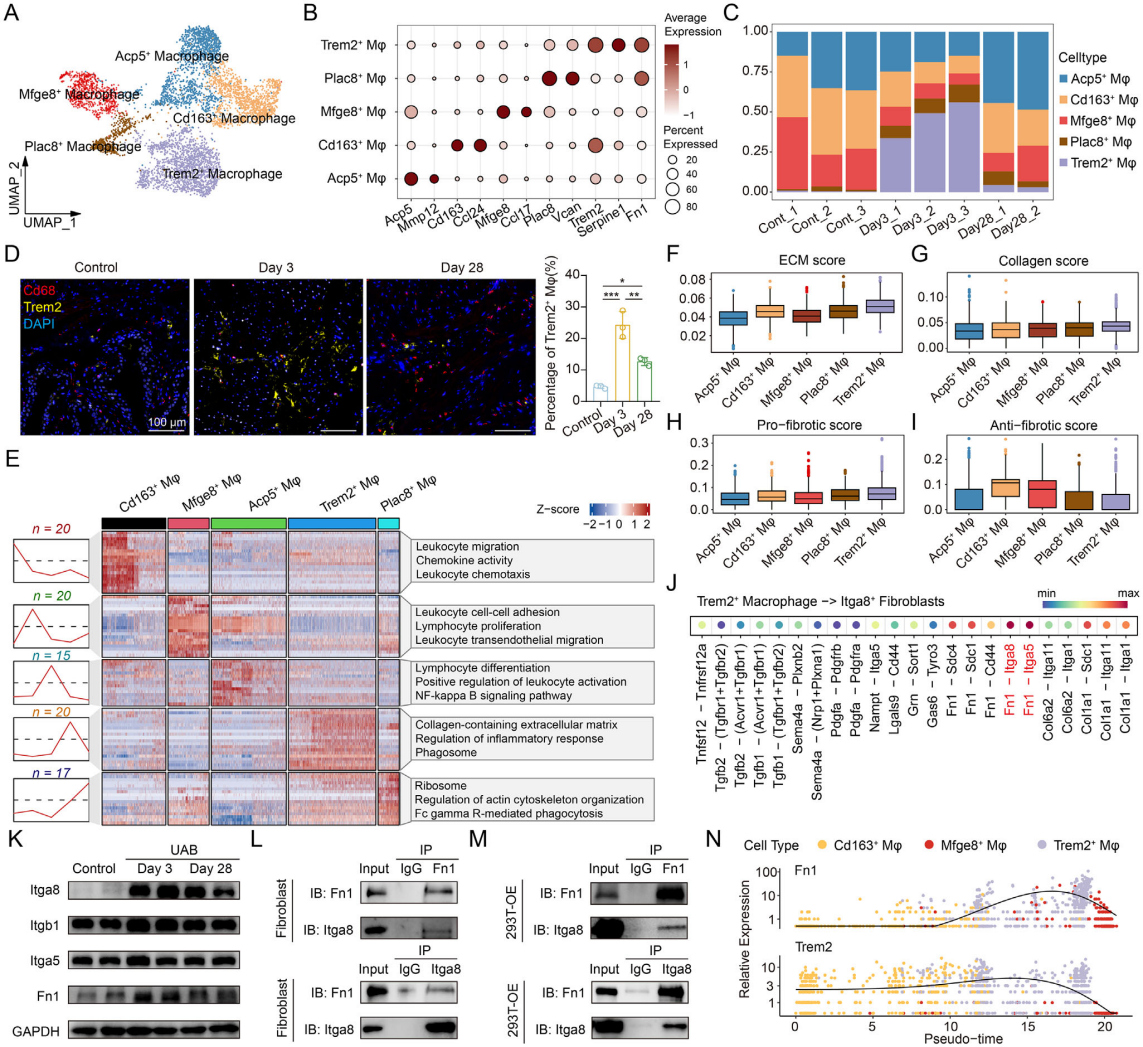
Trem2⁺ macrophages interact with fibroblasts via the Fn1‐Itga8 ligand‐receptor pairing. A) UMAP plot of subclustered macrophages. B) Dot plot of markers for each subset. C) Frequency of each cluster at different time points. D) Representative immunofluorescence images and quantitative analysis of Cd68 (red), Trem2 (yellow), and DAPI (blue) expression in different groups, n = 3 per group. E) Representative GO and KEGG enrichment of the marker genes expressed in each subset of macrophages. F) Box plots of the ECM score for each subset. G) Box plots of the collagen score for each subset. H) Box plots of the pro‐fibrotic score for each subset. I) Box plots of the anti‐fibrotic score for each subset. J) Bubble plot showing the primary ligand‐receptor pairs involved in the interaction between Trem2⁺ macrophages and Itga8⁺ fibroblasts. K) Representative western blot images of Itga8, Itgb1, Itga5, and Fn1. L) Representative images of the immunoprecipitation assay in fibroblasts. M) Representative images of the immunoprecipitation assay in 293T cells overexpressing Itga8. N) Dot plots demonstrated the expression dynamics of *Fn1* and *Trem2* along pseudo‐time. Mφ = Macrophage. Data represent mean ± SD. One‐way ANOVA was used for D. ^***^ *p* < 0.001.

In the intricate pathophysiological landscape of fibrosis, macrophages are recognized for their remarkable plasticity, enabling them to modulate inflammatory responses and ECM remodeling in a context‐dependent manner. Following nerve injury, macrophages represented the most significantly expanded immune population within the bladder microenvironment (Figure 1H,I). To dissect their heterogeneity and potential roles, we reclustered the macrophages into five transcriptionally distinct subpopulations: Acp5⁺, Cd163⁺, Mfge8⁺, Plac8⁺, and Trem2⁺ macrophages (**Figure** **4**A,B). Notably, Trem2^+^ macrophages were prominently enriched during the acute phase of BPNI (Figure 4C; Figure S10A,B, Supporting Information), as confirmed by immunofluorescence (Figure 4D). Functional enrichment linked Trem2⁺ macrophages to collagen‐containing ECM, regulation of inflammatory response, and phagosome (Figure 4E). Also, Trem2^+^ macrophages exhibited the highest ECM and collagen gene signature scores, along with the greatest pro‐fibrotic potential and the lowest anti‐fibrotic activity among macrophage clusters (Figure 4F–I), implicating them as a potent driver of early fibrotic remodeling. Interestingly, ligand‐receptor interaction analysis revealed robust communication between Trem2⁺ macrophages and Itga8⁺ fibroblasts, with the Fn1–Itga8 and Fn1‐Itga5 pairs emerging as the strongest among all potential macrophage–fibroblast interactions (Figure 4J; Figure S10C–E, Supporting Information). However, Fn1 and Itga8 were upregulated in denervated bladder tissue, whereas Itga5 remained stable, hinting at a preferential role for the Fn1‐Itga8 interaction (Figure 4K). Co‐immunoprecipitation validated physical interactions between Fn1 and Itga8 in both bladder fibroblasts and Itga8‐overexpressing 293T cells, reinforcing that Fn1 serves as a functional ECM ligand mediating integrin signaling in fibrosis (Figure 4L,M). Pseudotime trajectory analysis further demonstrated that the emergence of Trem2⁺ macrophages coincided with dynamic upregulation of Fn1, underscoring their role in ECM remodeling and fibroblast activation (Figure 4N; Figure S10F–G, Supporting Information). Thus, Trem2⁺ macrophages orchestrated denervation‐induced bladder fibrosis by activating fibroblasts through the Fn1‐Itga8 axis.

**Figure 5:**
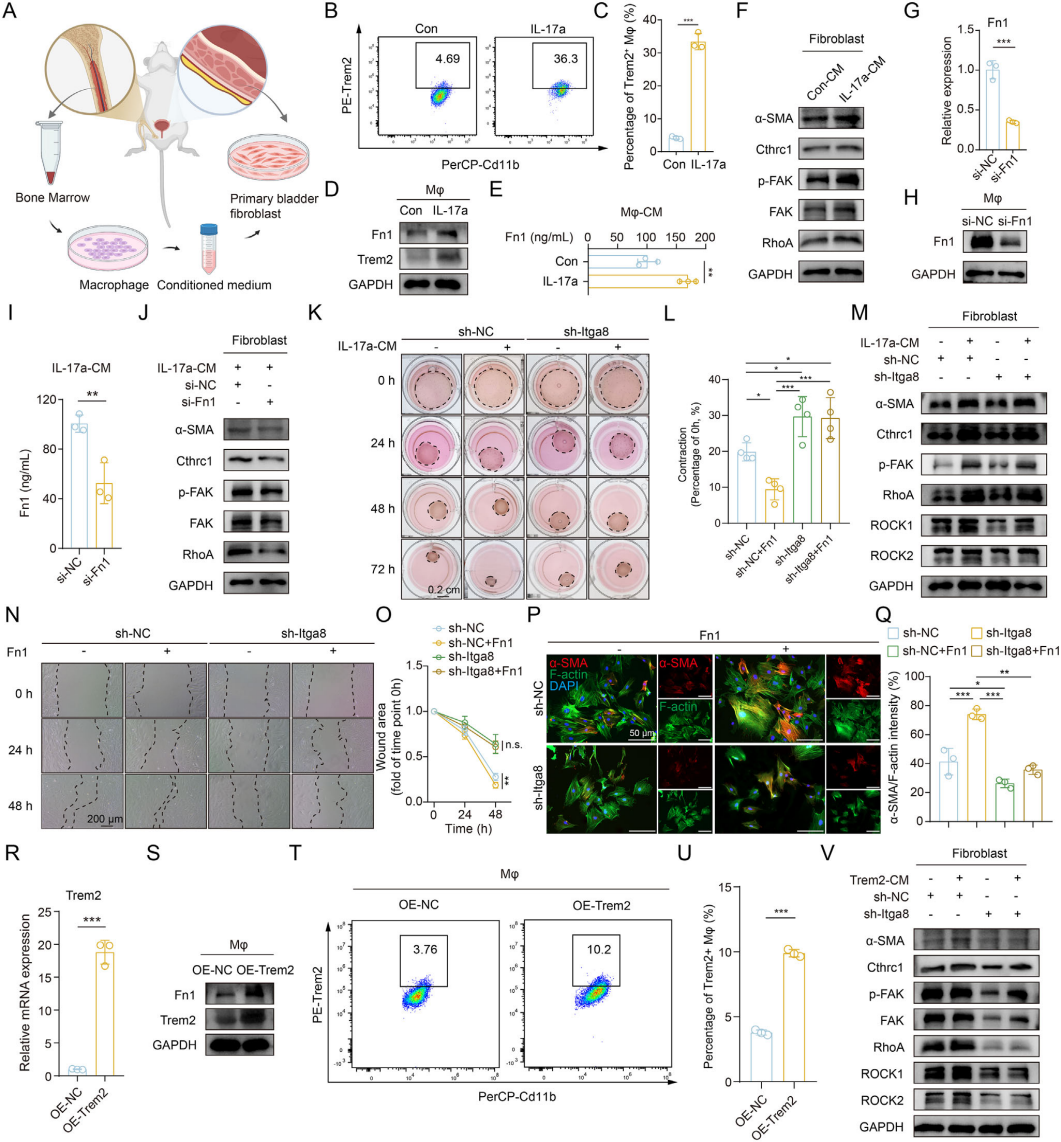
Co‐culture experiments demonstrate that Fn1 derived from Trem2⁺ macrophages activates fibroblasts via Itga8. A) Schematic representation of the co‐culture experiment design. B) Flow cytometry confirms that IL‐17a induces the differentiation of BMDM into Trem2⁺ macrophages in vitro. C) Quantification of the percentage of Trem2^+^ macrophage in (B), n = 3 per group. D) Representative Western blot images of in vitro‐induced Trem2⁺ macrophages. E) ELISA analysis of Fn1 expression in the conditioned medium. F) Representative Western blot images of primary bladder fibroblasts treated with conditioned medium from IL‐17a‐induced BMDM. G,H) qRT‐PCR (G) and Western blot (H) analysis of Fn1 expression at the mRNA and protein levels following transfection with Fn1 siRNA. I) ELISA analysis of Fn1 expression in the culture supernatant following transfection with Fn1 siRNA. J) Representative Western blot images of fibroblasts treated with conditioned medium from IL‐17a‐induced BMDM transfected with either si‐NC or si‐Fn1. K) Representative images of the contraction assay in fibroblasts transduced with sh‐NC or sh‐Itga8 lentivirus and treated with the conditioned medium. L) Quantification of cell contraction in (K), n = 4 per group. M) Representative Western blot images of fibroblasts transduced with sh‐NC or sh‐Itga8 lentivirus and treated with conditioned medium from IL‐17a‐induced BMDM. N) Representative images of the scratch assay in fibroblasts transduced with sh‐NC or sh‐Itga8 lentivirus and treated with Fn1. O) Statistical analysis of the wound area over time in (N), n = 3 per group. P) Representative images of immunofluorescence staining in fibroblasts transduced with sh‐NC or sh‐Itga8 lentivirus and treated with Fn1. Q) Quantification of α‐SMA/F‐actin in (P), n = 3 per group. R,S) qRT‐PCR (R) and Western blot (S) analysis of Trem2 expression at the mRNA and protein levels following transfection with OE‐Trem2. T) Flow cytometry confirms that IL‐17a induces the differentiation of BMDM into Trem2⁺ macrophages in vitro. U) Quantification of the percentage of Trem2^+^ macrophage in (T), n = 3 per group. V) Representative Western blot images of fibroblasts transduced with sh‐NC or sh‐Itga8 lentivirus and treated with conditioned medium from Trem2‐overexpressing BMDM. Mφ = Macrophage. Data represent mean ± SD. Unpaired two‐tailed t‐test was used for C, E, G, I, R, and U. One‐way ANOVA was used for L, and Q. Two‐way ANOVA was used for O. ^*^P < 0.05, ^**^P < 0.01, ^***^ *p* < 0.001. Figure 5A was created in BioRender. Wang, J. (2025) https://BioRender.com/kp68v7m.

To further explore the effect of macrophage‐fibroblast crosstalk, an in vitro co‐culture system comprising bone marrow‐derived macrophages (BMDMs) and primary bladder fibroblasts was established (**Figure** **5**A). Consistent with existing evidence implicating IL‐17a in macrophage polarization,^[^ ^24^ ^]^ IL‐17a robustly induced BMDM differentiation into Trem2^+^ macrophages (Figure 5B,C), accompanied by upregulation of Trem2 and Fn1 protein levels (Figure 5D) and increased Fn1 secretion (Figure 5E). Conditioned medium from IL‐17a‐treated BMDMs markedly enhanced the activation of primary fibroblasts, as evidenced by elevated α‐SMA and Cthrc1 expression, FAK phosphorylation, and RhoA upregulation (Figure 5F). To determine whether BMDM‐derived Fn1 contributes to fibroblast activation, we silenced Fn1 in BMDMs using siRNA and confirmed the knockdown efficiency at both mRNA and protein levels (Figure 5G,H), while ELISA showed a corresponding reduction in extracellular Fn1 (Figure 5I). Notably, Fn1‐knockdown BMDMs failed to fully activate fibroblasts, which showed reduced α‐SMA and Cthrc1 expression, as well as diminished FAK and RhoA activation (Figure 5J). Furthermore, conditioned medium from IL‐17a‐treated BMDMs significantly enhanced fibroblast contractility, which was abrogated upon Itga8 knockdown in fibroblasts (Figure 5K,L). Western blot (Figure 5M), scratch‐wound migration assays (Figure 5N,O), and immunofluorescence (Figure 5P,Q) consistently demonstrated that Itga8 silencing attenuated fibroblast activation induced by IL‐17a‐stimulated BMDMs. Additionally, overexpression of Trem2 in BMDMs led to upregulation of Fn1 at both transcript and protein levels (Figure 5R,S), along with expansion of the Trem2⁺ macrophage population (Figure 5T,U). Co‐culture with these Trem2‐overexpressing macrophages markedly enhanced fibroblast activation, which was mitigated by Itga8 depletion in fibroblasts (Figure 5V). Collectively, these findings provided strong evidence that Trem2^+^ macrophages promoted fibroblast activation via the Fn1‐Itga8 signaling, and that targeting Itga8 effectively disrupts this profibrotic crosstalk.

### 2.5 Macrophage Depletion Attenuates Neurogenic Bladder Fibrosis and Improves Function

**Figure 6:**
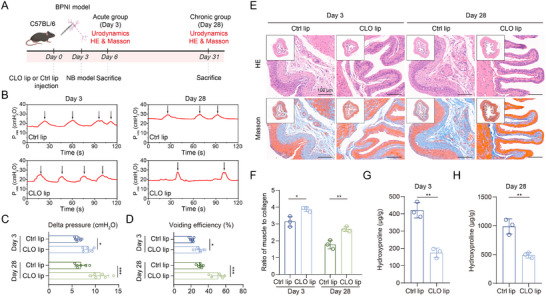
Macrophage depletion improves voiding function and ameliorates fibrosis in the neurogenic bladder. A) Schematic diagram of the animal experimental design. B) Representative urodynamic traces from each group at day 28 post‐BPNI. The black arrow indicates a single voiding event. C,D) Quantitative analysis of urodynamic parameters showing (C) delta pressure and (D) voiding efficiency (calculated as voided volume/maximum bladder capacity) in clodronate liposomes (CLO lip) and control liposomes (Ctrl lip) groups (n = 6 per group). E) Representative HE and Masson's trichrome staining of the bladder from each group. F) Quantification of the smooth muscle to collagen ratio from histology sections shown in (E) (n = 3 per group). G,H) Hydroxyproline content in bladder tissues from the CLO lip and Ctrl lip groups at (G) day 3 and (H) day 28 (n = 3 per group). Data are presented as mean ± SD. One‐way ANOVA was used for C, D, F. Unpaired two‐tailed Student's *t*‐test was used for G and H. ^*^ *p* < 0.01, ^**^ *p* < 0.01, ^***^ *p* < 0.001. Figure 6A was created in BioRender. Wang, J. (2025) https://BioRender.com/mmrjp3l.

To directly evaluate the contribution of macrophages to NB pathogenesis, we performed a depletion experiment using clodronate liposomes (CLO lip). Mice were pre‐treated with CLO lip or control liposomes (Ctrl lip) three days prior to BPNI induction (**Figure** **6**A). The efficacy of macrophage depletion was verified by immunofluorescence, which showed a significant reduction in macrophages in the CLO lip group at both acute and chronic phases (Figure S11, Supporting Information). Depletion of macrophages resulted in significant functional improvement at both 3 days and 28 days post‐injury, as demonstrated by enhanced detrusor contractility (delta pressure) and voiding efficiency compared to the control group (Figure 6B–D). Histological examination and biochemical analysis confirmed a marked attenuation of fibrotic remodeling, characterized by significantly reduced collagen deposition in Masson's trichrome staining (Figure 6E,F) and a corresponding decrease in hydroxyproline content upon macrophage ablation (Figure 6G,H). These data demonstrated that macrophages played a pivotal role in driving both the functional deficits and structural fibrosis in NB.

### 2.6 Local Itga8 Knockdown Mitigates Bladder Fibrosis and Improves Function in a Rat Model

**Figure 7:**
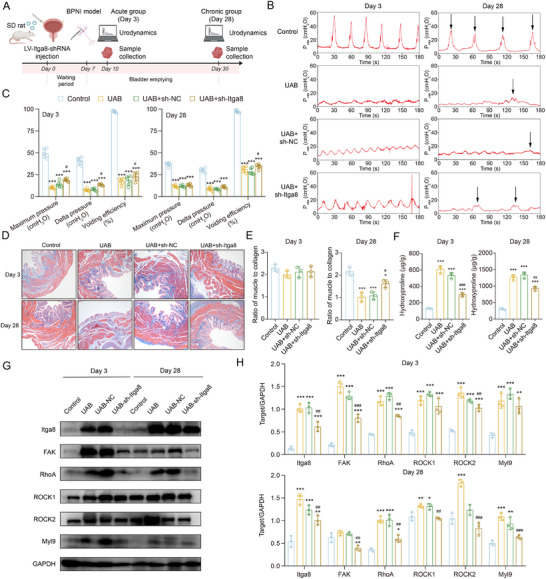
Knockdown of Itga8 improves urodynamic parameters and bladder fibrosis in rats. A) Schematic diagram of the animal experimental design. B) Representative urodynamic features during the acute and chronic phases of BPNI in the control, NB, NB + sh‐NC, and NB + sh‐Itga8 groups. The black arrow indicates a single voiding event. C) Bar graphs representing the maximum intravesical pressure, change in intravesical pressure, and voiding efficiency (calculated as voided volume/maximum bladder capacity) for each group, n = 6 per group. D) Representative images of Masson's trichrome staining for each group. E) Quantification of the ratio of smooth muscle to collagen in (E), n = 3 per group. F) Measurement of hydroxyproline content during the acute and chronic phase of BPNI, n = 3 per group. G,H) Representative western blot images (G) and quantitative analysis (H) of Itga8, FAK, RhoA, ROCK1, ROCK2, and Myl9. Data represent mean ± SD. One‐way ANOVA was used for C, E, F, and H. Compared with the control group, ^*^ *p* < 0.05, ^**^ *p* < 0.01, ^***^ *p* < 0.001. Compared with the sh‐NC group, ^#^ *p* < 0.05, ^##^ *p* < 0.01, and ^###^ *p* < 0.001. Figure 7A was created in BioRender. Wang, J. (2025) https://BioRender.com/3xlbk8m.

In light of Itga8's role in kidney development^[^ ^25^ ^]^ and with a view to mitigating the potential adverse effects, a localized gene silencing approach was employed. This strategy involved the direct injection of lentivirus encoding sh‐Itga8 or a non‐targeting control (sh‐NC) into the rat bladder wall, with the objective of evaluating the therapeutic efficacy in a living organism. To assess the specificity of this approach, we systematically analyzed the transduction efficiency across different cell types, demonstrating preferential lentiviral targeting of fibroblasts and the consequent reduction of Itga8 (Figure S12, Supporting Information). The intervention was administered 7 days before BPNI to achieve optimal knockdown efficiency (**Figure** **7**A). Urodynamic assessment revealed that sh‐Itga8 treatment significantly improved bladder dynamics during the acute phase, while NB + sh‐NC had no impact on voiding function compared to untreated NB rats. Specifically, maximal intravesical pressure, pressure change during voiding, and overall voiding efficiency were all enhanced in the NB + sh‐Itga8 group (Figure 7B,C). Although pressure‐related parameters showed no statistical improvement during the chronic phase, voiding efficiency remained consistently elevated, indicating sustained functional benefit. Masson's trichrome staining demonstrated a marked reduction in collagen deposition in sh‐Itga8‐treated bladders (Figure 7D,E), supported by decreased hydroxyproline content (Figure 7F). Histological analysis of heart, liver, spleen, lung, and kidney showed no off‐target toxicity, confirming the safety of localized Itga8 suppression (Figure S13, Supporting Information). Mechanistically, Itga8 silencing inhibited FAK/RhoA/ROCK signaling activation at both early and late post‐injury stages (Figure 7G,H). Overall, these data demonstrated that local Itga8 inhibition mitigates fibrotic remodeling and improves bladder urodynamics.

### 2.7 Fibroblast‐Specific Deletion of Itga8 Attenuates Bladder Fibrosis and Restores Function In Vivo

**Figure 8:**
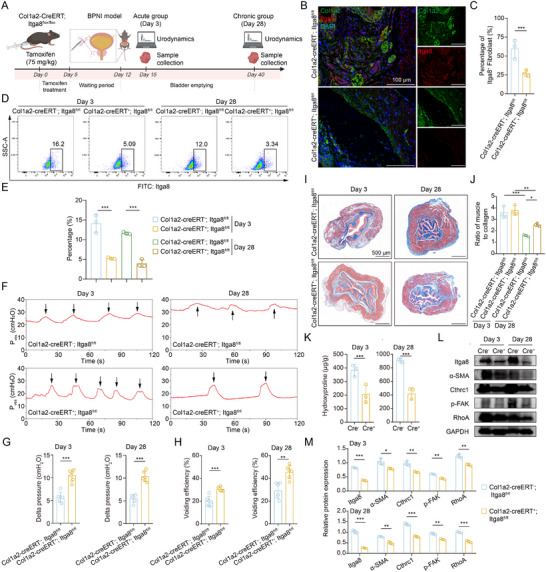
*Col1a2*‐CreERT; *Itga8* ^fl/fl^ mice demonstrates that Itga8 deletion delays fibrosis progression and protects bladder function. A) Schematic diagram of the animal experimental design. B) Representative immunofluorescence images of Col1a2 (green), Itga8 (red), and DAPI (blue) expression in different groups. C) Quantitative analysis of percentage of Itga8^+^ fibroblasts, n = 3 per group. D) Representative flow cytometry plots of Itga8^+^ fibroblasts in each group. E) Quantification of the percentage of Itga8^+^ fibroblasts in (D), n = 3 per group. F) Representative urodynamic profiles during the acute and chronic phases of BPNI in *Col1a2*‐CreERT⁺; *Itga8* ^fl/fl^ mice and their littermate controls. The black arrow indicates a single voiding event. G,H) Bar graphs representing (G) delta intravesical pressure and (H) voiding efficiency (calculated as voided volume/maximum bladder capacity) for each group, n = 6 per group. I) Representative images of Masson's trichrome staining for each group. J) Quantification of the ratio of smooth muscle to collagen in (I), n = 3 per group. K) Measurement of hydroxyproline content during the acute and chronic phase of BPNI, n = 3 per group. L,M) Representative western blot images (L) and quantitative analysis (M) of isolated fibroblasts from the acute and chronic phases of BPNI in *Col1a2*‐CreERT⁺; *Itga8* ^fl/fl^ mice and their littermate controls. Data represent mean ± SD. An unpaired two‐tailed *t*‐test was used for C, G, H, K, and M. One‐way ANOVA was used for E and J. ^*^ *p* < 0.05, ^**^ *p* < 0.01, ^***^ *p* < 0.001. Figure 8A was created in BioRender. Wang, J. (2025) https://BioRender.com/avquqdv.

To validate the cell‐autonomous role of fibroblast‐expressed *Itga8* in fibrosis, we generated *Col1a2*‐CreERT; *Itga8* ^fl/fl^ mice. This model was selected based on our scRNA‐seq data confirming exclusive *Col1a2* expression in fibroblasts and its co‐localization with *Itga8*, enabling precise tamoxifen‐inducible and fibroblast‐specific Itga8 deletion (Figure S14, Supporting Information). Furthermore, *Col1a2*‐CreERT; *Rosa26*‐tdTomato mice confirmed highly specific Cre‐mediated recombination within fibroblasts (Figure S15, Supporting Information). *Col1a2*‐CreERT; *Itga8* ^fl/fl^ mice received tamoxifen (75 mg kg^−1^) for five consecutive days, followed by a 7‐day waiting period to ensure efficient recombination before BPNI induction. (**Figure** **8**A; Figure S16A, Supporting Information). Immunofluorescence staining confirmed efficient and specific depletion of Itga8 in fibroblasts. (Figure 8B,C). Flow cytometry of bladder stromal cells (Cd45^–^Cd31^–^Epcam^–^) revealed a significant reduction in the Itga8⁺ fibroblast population in knockout animals, validating effective and selective targeting of fibroblasts (Figure 8D,E; Figure S16B, Supporting Information). Urodynamic analysis revealed that *Col1a2*‐CreERT^+^; *Itga8* ^fl/fl^ mice maintained significantly lower intravesical pressures and exhibited enhanced voiding efficiency at 3 and 28 days post‐BPNI compared to littermate controls (Figure 8F–H), providing compelling evidence for a sustained functional benefit of fibroblast‐specific *Itga8* deletion. Histopathological evaluation further substantiated the functional improvements that Itga8‐deficient mice exhibited reduced collagen accumulation (Figure 8I,J) and lower hydroxyproline content (Figure 8K). Moreover, fibroblasts isolated from *Col1a2*‐CreERT^+^; *Itga8* ^fl/fl^ mice demonstrated a concurrent downregulation of α‐SMA and Cthrc1 expression (Figure 8L,M). Consistent with the above, fibroblast‐specific Itga8 deletion led to suppressed FAK phosphorylation and RhoA signaling, indicating disruption of key profibrotic pathways activation (Figure 8L,M). Importantly, the absence of any detectable effect of *Itga8* deletion in healthy bladders ruled out nonspecific disruption of normal function and supports the conclusion that the observed therapeutic benefits are specifically attributable to the inhibition of its pathological signaling (Figure S17, Supporting Information). Taken together, these findings demonstrate that fibroblast‐specific Itga8 deletion attenuates fibroblast activation and fibrosis while restoring bladder function after denervation, establishing Itga8 as a therapeutic target for NB fibrosis.

## 3 Conclusion

**Figure 9:**
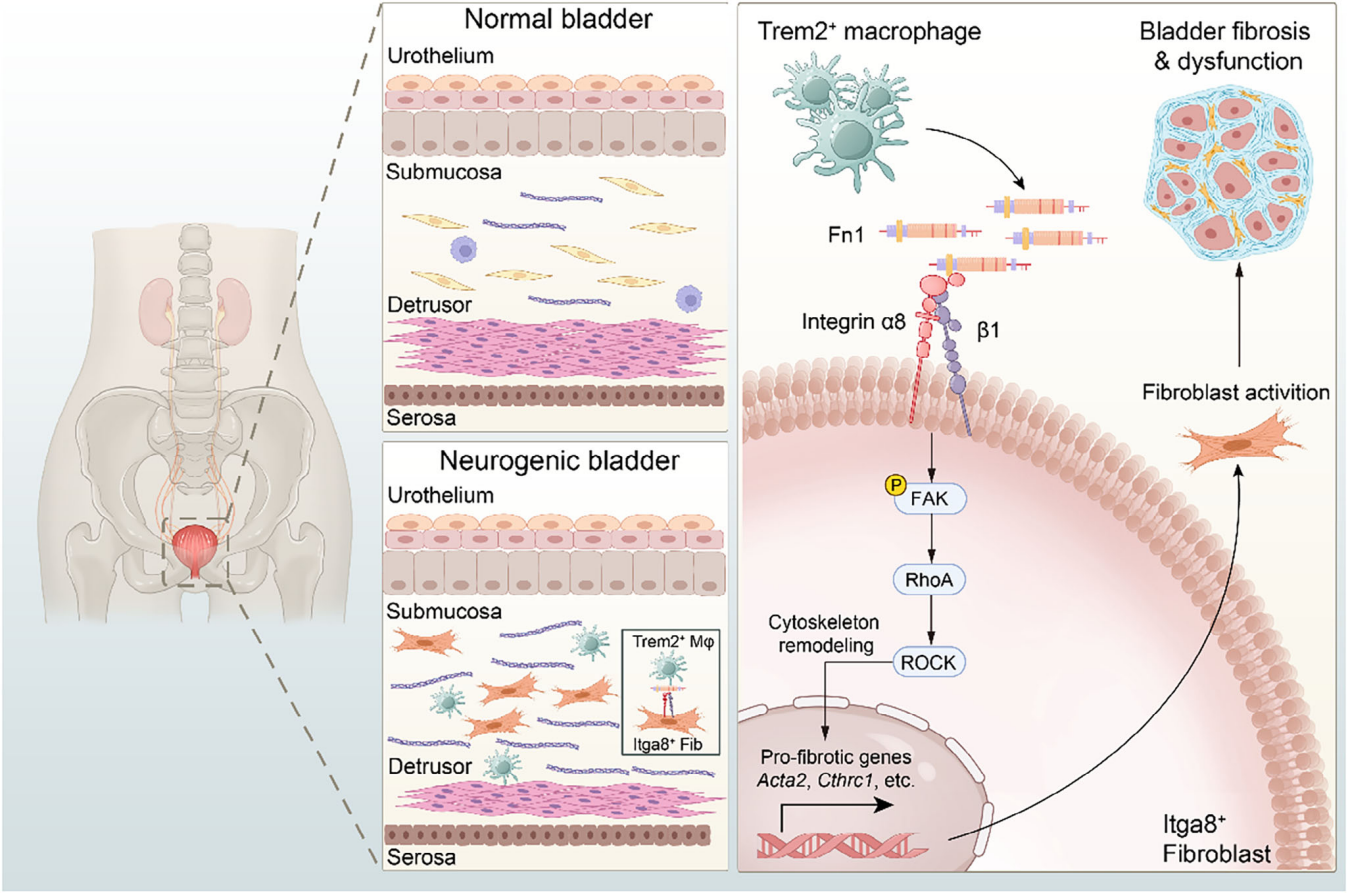
Schematic diagram for the process of Itga8^+^ fibroblast activation in the context of neurogenic bladder fibrosis. In the normal bladder, fibroblasts and macrophages were in a state of quiescence. In contrast, neurogenic bladder was characterized by a substantial augmentation in the population of Itga8⁺ fibroblasts during the acute phase post‐injury. Moreover, fibroblast activation was orchestrated by Trem2⁺ macrophages, which secreted Fn1 to engage Itga8 on fibroblasts, thereby reinforcing the pro‐fibrotic communication between fibroblasts and macrophages. This, in turn, activated the FAK/RhoA/ROCK pathway and increased the expression of pro‐fibrotic genes through cytoskeletal remodeling, leading to fibroblast activation and ultimately to bladder fibrosis. Inhibition of Itga8 was expected to block the pathways mentioned above, delay fibrogenesis, and preserve bladder function integrity. Created in BioRender. Wang, J. (2025) https://BioRender.com/m2gslzz.

Bladder fibrosis is characterized by ECM deposition, particularly collagen accumulation, ultimately leading to irreversible loss of bladder contractility and contributing significantly to the poor therapeutic outcomes in NB. Fibroblast activation is a central pathophysiological event in fibrosis; however, its underlying mechanisms in NB remain poorly defined. Leveraging scRNA‐seq, we identified a distinct Itga8⁺ fibroblast subpopulation that plays a pivotal fibrogenic role in bladder fibrosis. Mechanistically, Itga8 regulated fibroblast contractility and cytoskeleton remodeling through the FAK/RhoA/ROCK signaling pathway. Furthermore, Trem2⁺ macrophages, which were markedly expanded following nerve injury, instigated fibroblast activation through the Fn1‐Itga8 pro‐fibrotic axis. Critically, ablation of macrophages mitigated fibrotic remodeling and improved bladder function, establishing their critical involvement in the progression of NB. In vivo, both local shRNA‐mediated knockdown of Itga8 and fibroblast‐specific genetic ablation of Itga8 significantly attenuated fibrosis progression and improved urodynamic parameters (**Figure** **9**). Collectively, our findings uncovered a critical role for Itga8⁺ fibroblasts in the pathogenesis of bladder fibrosis and suggested that therapeutic targeting of Itga8 may offer a novel strategy to preserve bladder function in NB.

Fibrosis is a hallmark pathological feature of late‐stage NB, manifesting as reduced bladder compliance secondary to excessive ECM deposition.^[^ ^10^ ^]^ Fibroblasts, owing to their intrinsic plasticity and capacity for phenotypic transitions, constitute the primary cellular source of ECM in the fibrotic process. In response to mechanical stress, chemical cues, and inflammatory stimuli, fibroblasts can be activated into myofibroblasts, acquiring enhanced contractile and ECM synthesis capabilities.^[^ ^8^ ^]^ For instance, TGF‐β, a classic fibrotic cytokine, has been demonstrated to phosphorylate the intracellular Smad2/3 transcription factors, thereby inducing fibroblast activation and collagen production.^[^ ^26^ ^]^ In this study, we found that the TGF‐β/Smad pathway was activated as early as 3 days post‐neural injury, suggesting that the initiation of fibrosis occurs earlier than expected. Recent research has also supported that bladder fibrosis induced by spinal cord injury occurs much earlier than that induced by bladder outlet obstruction.^[^ ^27^ ^]^ This observation suggests that bladder fibrosis resulting from nerve injury requires more in‐depth attention and prompt intervention. Therefore, a more precise characterization of the heterogeneity and phenotypes of fibroblasts following nerve injury will facilitate the early implementation of rational, highly targeted anti‐fibrotic interventions. Using scRNA‐seq, we characterized the transcriptional landscape of bladder fibroblasts post‐denervation and identified robust activation and marked heterogeneity during the early stages of fibrosis. This heterogeneity included a rare Upk3a⁺ fibroblast subset, and the potential contribution of mechanisms such as EMT to the expansion or phenotypic diversification of fibroblast populations in this context warrants further investigation. Notably, a distinct subpopulation, Itga8⁺ fibroblasts, emerged as a dominant subset in the initial stages of denervation‐induced fibrosis. Itga8⁺ fibroblasts displayed transcriptional profiles indicative of increased collagen secretion, cytoskeletal changes, and contractile properties, which were hallmark features of activated fibroblasts.^[^ ^28^ ^]^ Together, these findings highlighted Itga8⁺ fibroblasts as key mediators of ECM accumulation and impaired bladder compliance.

Itga8, a member of the integrin family of transmembrane adhesion receptors, plays a critical role in transducing biochemical and biomechanical signals between cells and their microenvironment under both physiological and pathological conditions.^[^ ^29^ ^]^ In mammals, integrin receptors are constituted as heterodimers comprising α‐ and β‐subunits. Through the interaction with extracellular ligands, they regulate cytoskeletal structure, intracellular signaling, and gene expression. The classification of integrins is determined by the recognition motif, with all eight members of the Arg‐Gly‐Asp (RGD) ‐binding family exhibiting the capacity to recognize the amino acid sequence in their RGD ligands, including Itga8. These integrins bind to ECM components containing RGD, such as Fn1, osteopontin, vitronectin, and fibrinogen, thereby activating intracellular signaling pathways. Previous studies have established that integrin engagement activates multiple downstream pathways such as FAK,^[^ ^30^ ^]^ PI3K/Akt,^[^ ^31^ ^]^ and Src/MAPK,^[^ ^32^ ^]^ thereby modulating cellular behavior. Here, we demonstrated that Itga8 mediates fibroblast activation through the FAK/RhoA/ROCK axis, which was crucial for regulating cytoskeletal dynamics and enhancing cellular contractility. FAK, a pivotal effector downstream of integrin signaling, has been shown to exhibit kinase activity and, in turn, facilitate the recruitment or phosphorylation of other focal adhesion (FA) proteins.^[^ ^33^ ^]^ In addition, FAK facilitates the localization and cyclic activation of guanine nucleotide exchange factors (GEFs) and GTPase‐activating proteins (GAPs), thereby enabling the spatial and temporal regulation of RhoGTPase activity,^[^ ^34^ ^]^ further contributing to activation of RhoA/ROCK and subsequent remodeling of the cytoskeleton. Furthermore, RhoA/ROCK signaling can be transmitted to the nucleus via the linker of nucleoskeleton and cytoskeleton(LINC) complex, leading to the upregulation of profibrotic genes, such as plasminogen activator inhibitor‐1 and connective tissue growth factor.^[^ ^35^, ^36^ ^]^ Overall, our findings provided direct evidence that Itga8 mediates fibroblast activation through the FAK/RhoA/ROCK signaling axis, linking integrin engagement to intracellular signaling pathways that drive fibrosis.

Given the broad role of integrin receptors in regulating cell polarity, cytoskeletal dynamics, and TGF‐β regulation, the development of anti‐fibrotic drugs targeting integrins has garnered substantial interest. Several drugs targeting either specific integrin receptors or broad integrin sites have been explored clinically in fibrosis‐related diseases such as idiopathic pulmonary fibrosis (IPF) and primary sclerosing cholangitis (PSC).^[^ ^22^ ^]^ Bexotegrast (PLN‐74809), a dual‐selective inhibitor of αvβ6 and αvβ1 integrins, resulted in a significant reduction in 68Ga‐CBP8 uptake and decreased extravascular extracellular volume, suggesting potential antifibrotic effects and favorable lung remodeling in patients with IPF.^[^ ^37^ ^]^ Several ongoing phase II clinical trials are evaluating the effective dose of Bexotegrast in PSC (NCT04480840) and acute respiratory distress syndrome (NCT04565249). Moreover, preliminary phase I clinical trials have confirmed the satisfactory tolerability of the integrin αvβ6‐targeting pharmaceutical agent GSK3008348 in patients suffering from IPF, with no serious adverse events.^[^ ^38^ ^]^ The safety, pharmacokinetics, and immunogenicity of BG00011 (former name: STX‐100), a humanized monoclonal antibody targeting αvβ6 integrin in IPF patients, is being evaluated (NCT01371305). Compared to the potential side effects on multiple organs caused by global inhibition of TGF‐β, blocking integrins (especially anti‐integrin αv) has been demonstrated to be an effective anti‐fibrotic treatment with fewer associated adverse reactions.^[^ ^39^ ^]^ Notably, from an evolutionary perspective, phylogenetic trees reveal that Itga8 shares structural similarities with Itgav subunits.^[^ ^40^ ^]^ Both Itga8 and Itgav are RGD‐binding integrins that bind the same ECM components, such as Fn1, osteopontin, and vitronectin.^[^ ^25^ ^]^ Targeting Itga8 has been found to improve diseases like non‐alcoholic steatohepatitis and renal interstitial fibrosis.^[^ ^41^, ^42^ ^]^ Similarly, our in vivo experiments demonstrated that genetic suppression of Itga8 expression significantly ameliorated bladder fibrosis and urodynamic function. Therefore, these findings confirmed that Itga8 is a promising therapeutic target for bladder fibrosis. Focusing on the development of monoclonal antibodies or small molecule inhibitors with high specificity for Itga8 could significantly enhance its translational potential as a precision therapeutic strategy for bladder fibrosis.

In addition to fibroblasts, accumulating studies have highlighted the significant involvement of immune cells, particularly macrophages, in tissue fibrosis. Our data demonstrated that macrophage depletion using clodronate liposomes preserved bladder function and attenuated fibrosis, underscoring their indispensable contribution to NB. This finding aligns with the established paradigm that macrophages orchestrate the initiation and progression of fibrotic processes.^[^ ^43^ ^]^ Recent studies have deepened our mechanistic understanding of macrophage‐driven fibrosis, including the elucidation of specific pathways such as the PRDX4‐PTEN axis in alveolar macrophages that promote fibroblast‐myofibroblast transition.^[^ ^44^ ^]^ Furthermore, GPNMB^+^ macrophages have been shown to foster a profibrotic microenvironment via GPNMB‐ITGB5 interactions in vascular fibrosis.^[^ ^45^ ^]^ Notably, macrophage‐specific mechanotransduction ablation has been reported to suppress profibrotic transcriptional programs in fibroblasts and mitigate scar formation.^[^ ^46^ ^]^ Together, these insights highlight the importance of defining and therapeutically targeting the specific macrophage subpopulations that drive NB‐associated fibrosis.

Trem2, a transmembrane receptor originally identified in neurodegenerative diseases, is primarily expressed on myeloid cells and is implicated in the regulation of immune response.^[^ ^47^ ^]^ Recent studies have elucidated additional roles of Trem2 in lipid metabolism, scar formation, and fibrosis progression. Trem2 regulates macrophage‐driven inflammation, lipid metabolism, and plaque stability, offering potential for therapeutic targeting in obesity related cardiovascular diseases.^[^ ^48^ ^]^ In IPF, alveolar macrophages exhibit elevated Trem2 expression and contribute to disease progression; conversely, Trem2 blockade alleviates fibrosis by reversing sphingomyelin‐induced macrophage dysfunction.^[^ ^49^ ^]^ Additionally, Trem2⁺ macrophages have been characterized as scar‐associated macrophages in endometriosis, where they display fibrosis‐promoting phenotypes.^[^ ^50^ ^]^ In the present study, using intercellular communication analysis and co‐culture experiments, we identified a key paracrine interaction between Trem2⁺ macrophages and fibroblasts mediated by the Fn1‐Itga8 ligand‐receptor axis, which was functionally involved in promoting fibroblast activation. Fn1 is a large multi‐domain protein that is widely present in the ECM, and its expression is often increased during tissue development, pathological progression, and wound healing.^[^ ^51^ ^]^ In fibrosis, the interaction between the EDA domain of Fn1 and integrin receptors has been demonstrated to contribute to disease progression by promoting cell contraction.^[^ ^52^ ^]^ In multiple sclerosis, an increase in Fn1 secretion by CD14^+^ pulmonary macrophages was observed, accompanied by fibrosis‐promoting characteristics.^[^ ^53^ ^]^ Moreover, macrophages after myocardial infarction were a major source of fibrosis‐enhancing factors like Fn1.^[^ ^54^ ^]^ Consistent with our findings, these studies suggested that macrophages may contribute to fibrotic remodeling by producing fibrotic matrix proteins. Therefore, targeting the Fn1‐Itga8 axis or modulating the activation of Trem2⁺ macrophages could represent novel therapeutic strategies for mitigating fibrosis. However, it is important to acknowledge the emerging context‐dependent roles of Trem2⁺ macrophages in fibrotic disease. In models of hepatic and dermal fibrosis, Trem2 deficiency has been associated with exacerbated fibrosis, indicating a potentially protective function in certain tissue environments and disease stages.^[^ ^55^, ^56^ ^]^ Future research should focus on elucidating the upstream signals that govern Trem2⁺ macrophage polarization in bladder fibrosis and further dissect the regulatory mechanisms underlying macrophage‐fibroblast crosstalk.

Although our study provided compelling evidence supporting Itga8 as a therapeutic target, several limitations should be acknowledged. First, the upstream molecular mechanisms responsible for the induction of Itga8 expression in fibroblasts remain unclear. Likewise, the signals that govern the differentiation of Trem2⁺ macrophages following neural injury warrant further investigation. Moreover, given that NB is a functional disorder of the lower urinary tract, access to human bladder tissue specimens is inherently limited, which constrains the extent of clinical validation.

In conclusion, our study identified Itga8^+^ fibroblasts as a key fibrogenic subset in NB and uncovered a central role of Itga8 in orchestrating ECM remodeling and pro‐fibrotic signaling. Therapeutic targeting of Itga8 could effectively delay fibrotic progression and preserve bladder function, providing a promising and potentially translatable therapeutic approach for NB.

## 4 Experimental Section

### Animals

The animal experiments involved in this study adhere to the Animal Research: Reporting of In Vivo Experiments (ARRIVE) guidelines. Experimental animals were obtained from the Experimental Animal Center of Tongji Hospital, Tongji Medical College, Huazhong University of Science and Technology, and housed in a specific pathogen‐free (SPF) facility. The animal experimental protocol was approved by the Tongji Hospital Experimental Animal Welfare Ethics Committee (TJH‐202207004).

Male SD rats, 8‐week‐old, were provided by Hunan SJA Laboratory Animal Co., Ltd. The *Col1a2*‐CreERT; *Itga8* ^fl/fl^ mice were generated by Cyagen Bioscience (Suzhou, China). A representative gel image of the genotype identification for *Col1a2*‐CreERT; *Itga8* ^fl/fl^ mice is shown in Figure S16A (Supporting Information). DNA was extracted from the mice's toes. The samples were incubated in 0.1 m sodium hydroxide at 98 °C for 1 h to allow complete cell lysis. After incubation, 1 m Tris‐HCl was added to neutralize the solution, followed by centrifugation at 12 000 rpm. The supernatant containing the DNA was collected for subsequent PCR analysis. The primer sequences for PCR are shown in Figure S16 (Supporting Information).

### BPNI Model

The establishment of the BPNI model is as described before.^[^ ^57^ ^]^ Briefly, anesthesia was induced using 2% isoflurane, and a midline infrapubic incision ≈2–3 cm in length was made to expose the bladder and prostate. The pelvic nerves were identified and clamped for 15 s with Dumont #5 forceps, and this procedure was repeated three times. Following the surgery, the animals were placed on a warming pad for recovery. Abdominal compression was applied twice daily to assist with urination post‐procedure.

### Urodynamics

The rodents were prepared by shaving the skin and performing disinfection, followed by a midline laparotomy under 1.5% isoflurane anesthesia to expose the bladder. A purse‐string suture was placed at the apex of the bladder, and a cystostomy was created. A bladder pressure measurement catheter (PE‐50) was inserted and secured. For *Col1a2*‐CreERT; *Itga8* ^fl/fl^ mice, cystometric analysis was performed using PE‐10 catheters to measure intravesical pressure. The abdominal cavity was closed in layers, and the catheter was exteriorized through the interscapular region. The catheter was connected to a BL‐420N biosignal acquisition and analysis system (Techman, Chengdu, China) and a microinfusion pump (LSP‐01‐1A, LongerPump, Baoding, China), ensuring that no air bubbles were present in the system.

Following a 2 h recovery period in a metabolic cage, the bladder was emptied via the catheter. The BL‐420N system software was activated, and the microinfusion pump was set to infuse prewarmed saline into the bladder at a rate of 100 µL min^−1^ (for *Col1a2*‐CreERT; *Itga8* ^fl/fl^ mice, the infusion rate was adjusted to 10 µL min^−1^) for 20 min, with 10 min rest intervals between infusions. Changes in bladder pressure and urine output were recorded.

### HE Staining

Paraffin sections were dewaxed and rehydrated through a series of graded solutions, while frozen sections were re‐warmed and fixed. Sections were pretreated with an HD constant staining pretreatment solution, followed by staining with hematoxylin for 3–5 min. Differentiation and bluing were then performed before staining with eosin for 15 s. Subsequently, the sections were dehydrated through a graded ethanol series, cleared with butanol and xylene, and mounted with neutral gum. Microscopic examination was conducted using a Nikon Eclipse E100 (Japan), followed by image acquisition and analysis.

### Masson Staining

The experiment was completed using the Masson dye solution set (G1006, Servicebio, Wuhan, China). The Masson staining protocol involved immersing paraffin sections in Masson A overnight, followed by a series of staining steps using a 1:1 mixture of Masson B and C. Differentiation was achieved with 1% hydrochloric acid alcohol, followed by treatments with solutions D, E, and F. After staining, the slides were rinsed with 1% glacial acetic acid, dehydrated in anhydrous ethanol, cleared with xylene, and mounted with neutral gum. Finally, microscopic examination was performed using a Nikon Eclipse E100 (Japan), followed by image acquisition and analysis.

### Hydroxyproline Detection

Hydroxyproline content was measured according to the standard procedure provided by the reagent supplier (BC0255, Solarbio, Beijing, China). Briefly, the procedure for determining hydroxyproline content in bladder tissue involved preparing standard solutions at various concentrations. Bladder tissue samples were weighed, cut, and incubated in an extraction solution until fully dispersed. After cooling and pH adjustment, the samples were centrifuged, and the supernatant was collected for analysis. The reaction system was set up according to the specified protocol, with reagents added to create both blank and test samples. The mixture was incubated, and absorbance was measured at A560 after cooling. A standard curve was constructed using the absorbance values of the standards, and the hydroxyproline content in the sample was calculated based on the sample weight and the dilution factor, yielding the concentration of hydroxyproline in the tissue sample.

### Functional Assessment of Detrusor Muscle Contractility

The bladder was rapidly excised and placed in Krebs' solution. After cleaning and removing the serosal and mucosal layers under a stereomicroscope, a detrusor muscle strip (≈10 mm × 3 mm × 1 mm) was carefully cut from the anterior wall. The strip was tied at both ends with 5–0 silk sutures and equilibrated in Krebs' solution for 30 min. For testing, one end of the strip was attached to a tension transducer, while the other end was fixed in an organ bath containing oxygenated Krebs' solution (95% O_2_, 5% CO_2_) at 37 °C. After a 30 min stabilization period, the tension was adjusted to 0.75 g and allowed to stabilize for an additional 30 min. Acetylcholine (ACh, 10^−5^ mol L^−1^) was added to the bath, and the contractile response of the detrusor strip was recorded.

### Single‐Cell RNA Sequencing (scRNA‐seq)

scRNA‐seq was performed by LC‐BIO TECHNOLOGIES (HANGZHOU) CO., LTD. Fresh bladder tissue was collected and prepared into a single‐cell suspension. After adjusting the cell concentration to the appropriate density, single‐cell sequencing libraries were constructed using the Chromium Single Cell 3′ Reagent Kits (10x Genomics). After passing library quality control, high‐throughput sequencing was performed on the Illumina sequencing platform using paired‐end sequencing. Raw data from sequencing were first processed using CellRanger v7.0.0, which included splitting and generating FASTQ files, aligning sequencing reads to the Rattus_norvegicus.Rnor_6.0 reference genome, filtering, and UMI counting. The gene‐barcode matrix was generated using cell barcodes. Subsequently, the data were normalized using the CellRanger Aggr function, resulting in a gene expression matrix for each cell.

### Data Filtering and Quality Control

Genes that were expressed in fewer than three cells were filtered out using Seurat v4.4.0.^[^ ^58^, ^59^ ^]^ Cells expressing fewer than 100 genes were also excluded to remove low‐quality cells, such as empty droplets or damaged cells. Cells with a high proportion of mitochondrial or red blood cell genes were filtered to remove stressed or dead cells. Furthermore, contamination and abnormal expression of marker genes in individual cells were removed using decontX v1.4.1, with a filtering threshold set to the recommended 0.2.^[^ ^60^ ^]^ Data were normalized using Seurat's default LogNormalize algorithm. High‐variable genes were identified using the FindVariableFeatures function, with the “features” parameter set to 3000. UMAP was used to visualize the cells based on the top 20 principal components.

### Marker Gene Analysis and Cell Annotation

Marker gene analysis was conducted using Seurat's built‐in FindAllMarkers function to identify genes highly expressed in specific clusters compared to all other cells. Differentially expressed genes (DEGs) were computed using the Wilcoxon rank‐sum test, and p‐values were adjusted using the Bonferroni correction. The following criteria were used to select marker genes: (1) gene expression present in >10% of cells in the target or control subgroups, (2) log(Fold Change) ≥ 0.26, (3) p ≤ 0.01. The cell annotation was performed by combining marker genes from the literature and the cellmarker2.0 database. Gene expression was visualized using FeaturePlot, and cell clusters were annotated accordingly. DotPlot was used to visualize the annotated results and the marker genes used.

### Subpopulation Analysis

The ClusterGVis v0.1.2 package was used to visualize the top 20 differential genes of each cell type in heatmaps, followed by biological process (BP) enrichment analysis to obtain functional pathways.^[^ ^61^ ^]^ For the subclustering of fibroblasts and macrophages, the respective cell populations were subset from the integrated object, and the standard Seurat workflow (including normalization, variable feature selection, scaling, PCA, and clustering) was reapplied to each subset. Fibroblast subclustering was performed using a graph‐based clustering algorithm on the top 20 principal components with a resolution parameter of 0.5. To define marker genes for these subpopulations, the FindAllMarkers function was used with the Wilcoxon rank‐sum test. Genes were considered definitive markers if they were detected in >10% of cells within the subpopulation, exhibited an average log2‐fold change ≥ 0.26, and achieved an adjusted p‐value ≤ 0.01. The resulting top marker genes, ranked by statistical significance and fold change, were used for functional annotation of the subpopulations.

### Gene Expression Correlation Analysis

Correlation analyses between gene expressions were performed on the log‐normalized expression data extracted from the Seurat object (slot = “data”). Pearson's correlation coefficient was calculated, and the statistical significance (*p*‐value) of the correlation was assessed using the cor. test function in R.

### Fibroblast hdWGCNA

The hdWGCNA v0.4.05 package was used to analyze the fibroblast subpopulations.^[^ ^21^ ^]^ Genes expressed in at least 5% of the cells were selected to construct co‐expression modules. A soft‐thresholding power of k = 25 was used, and the maximum overlap between module cells was set to 10 using the max_shared parameter. The TestSoftPowers function was employed to perform a parameter sweep for different soft‐thresholding powers. The optimal soft threshold was selected based on the scale‐free topology criterion. The results were visualized using PlotSoftPowers. Module expression on UMAP plots was displayed using the ModuleFeaturePlot function, while the top 35 genes with 500 co‐expression links were visualized using the ModuleNetworkPlot function. Gene‐module enrichment was performed using the top 650 genes from each module, and spatial gene coordinates were derived based on the module scores.

### Cell Communication

Fibroblasts and macrophages were merged using the merge function, and cell–cell communication was analyzed using CellChat v1.6.1.^[^ ^62^ ^]^ The Secreted Signaling, ECM‐Receptor, and Cell–Cell Contact databases were applied. Communication strength and quality between subpopulations were visualized using the netVisual_circle function, and communication bubble plots were generated using the netVisual_bubble function.

### Pseudotime Analysis

Pseudotime analysis was performed using the monocle v2.34.0 package to infer cell differentiation.^[^ ^63^ ^]^ Genes required for pseudotime inference were identified using Seurat's built‐in FindMarker function. Based on differentiation progress, cell subtypes were ordered, and gene sets were clustered into 50 clusters using ClusterGVis. Two clusters that showed significant changes in gene expression during differentiation were selected for further analysis. Gene set enrichment results were visualized using GSVA v2.0.7.^[^ ^64^ ^]^

### Gene Set Scoring

Following the approach described by Zhang et al., who calculated the ECM gene signature score using genes associated with the ECM (GO:0030198) from the Gene Ontology resource.^[^ ^65^ ^]^ The gene sets used for calculating collagen scores, myeloid cell clusters (pro‐fibrotic score, anti‐fibrotic score), and functional scores were adapted from published studies^[^ ^66^, ^67^ ^]^ and are summarized in Table S1 (Supporting Information). To calculate the gene feature score for each cell, the normalized expression matrix of genes included in a given gene set was used, and the average expression of all genes within the set was computed as the gene feature score for each cell.

### Immunofluorescence

The experiment was performed following the standard immunofluorescence protocol provided by the reagent manufacturer (G‐1255, Servicebio, Wuhan, China). Tissue sections were deparaffinized and rehydrated through sequential immersion in an environmentally friendly dewaxing solution and ethanol, followed by washing with distilled water. Antigen retrieval was performed under specified conditions, and sections were then washed with PBS. After circumscribing and sealing the sections, hydrogen peroxide was applied to quench endogenous peroxidase activity. Following serum blocking, primary antibodies were applied and incubated overnight at 4 °C. After washing, HRP‐conjugated secondary antibodies were added and incubated at room temperature. TSA amplification was applied, and following incubation, the slides were washed. Microwave treatment was used for antigen retrieval, and the process was repeated for multiple rounds of primary and secondary antibody applications. DAPI was used for nuclear staining, and tissue autofluorescence was quenched. The slides were then sealed with an anti‐fade mounting medium, and images were acquired using appropriate fluorescence wavelengths and filters (Pannoramic MIDI, 3DHISTECH, Hungary). The average fluorescence intensity and cell proportions were analyzed using ImageJ (v1.5.1, NIH, USA) software. The fluorescence intensity was quantified by measuring the mean gray value of the selected regions of interest (ROIs), and the cellular proportion was calculated based on the number of positively stained cells relative to the total number of cells in the field. The antibodies used in this study are listed in Table S2 (Supporting Information).

### RNA‐seq

Total RNA was isolated from transfected fibroblasts using TRIzol reagent (Invitrogen, Carlsbad, CA, USA), and its quality and quantity were assessed with a NanoDrop One (ThermoFisher, Waltham, MA, USA) and the Qubit RNA HS Assay Kit (Life Technologies, Invitrogen, USA). mRNA was captured with magnetic beads and converted into cDNA using M‐MuLV reverse transcriptase. The cDNA was then processed for sequencing library preparation, which included double‐stranded cDNA purification, addition of sequencing adapters, and ligation of UID adapters. The library was purified, and its quality was confirmed on the Agilent 4200 TapeStation before sequencing on the Illumina PE150 platform. Raw fastq data underwent quality control, including trimming, filtering, and removal of duplicate reads using UID labels. Gene expression levels were quantified using the FPKM method, and differential expression analysis was performed using the DESeq R package, identifying genes with significant changes in expression. Functional analysis of the differentially expressed genes (DEGs) was conducted using the clusterProfiler R package, focusing on pathways significantly enriched among the DEGs. Gene Set Enrichment Analysis (GSEA) was then performed using the clusterProfiler R package (version 3.8.0), with thresholds set as adjusted *p* value < 0.05 and FDR < 0.25.

### Western Blotting

Bladder tissue or cultured cells were homogenized in RIPA lysis buffer (AR0105, Boster, Wuhan, China) supplemented with protease inhibitors (HY‐K0010, MCE, Princeton, NJ, USA), followed by centrifugation to collect the supernatant for protein concentration determination using the BCA assay (AR0146, Boster, Wuhan, China). For western blot analysis, 30 µg of protein from each sample was electrophoresed and transferred onto PVDF membranes. After blocking with bovine serum albumin, the membranes were incubated with primary antibodies, followed by HRP‐conjugated secondary antibodies. Protein detection was performed using the ChemiDoc MP system (Bio‐Rad, Hercules, CA, USA), and band intensities were normalized to a reference protein and quantified using ImageJ software (v1.5.1, NIH, USA). The antibodies used in this study are listed in Table S2 (Supporting Information).

### Isolation of Primary Bladder Fibroblasts

SD rats aged 4–6 weeks were sacrificed by cervical dislocation, and the bladders were excised and rinsed in PBS containing 1% antibiotics. The bladders were then minced and digested with 2 mg mL^−1^ type I collagenase at 37 °C for 2 to 4 h, or until no visible tissue clumps remained. The cells were subsequently washed and resuspended in complete high‐glucose DMEM (supplemented with 15% FBS and 1% antibiotics) and cultured in T25 flasks. Cell identity was confirmed by immunofluorescence staining for collagen I and collagen III, and the cells tested negative for mycoplasma contamination.

### Lentivirus Production and Construction

Lentivirus construction was carried out by Genomeditech (Shanghai) Co., Ltd. To manipulate Itga8 expression, lentiviral vectors for Itga8 overexpression (OE‐Itga8) and knockdown (sh‐Itga8) were constructed. For sh‐Itga8, three effective shRNA sequences targeting Itga8 were selected and cloned into the PGMLV‐SC5 vector. The full‐length Itga8 coding sequence was inserted into a similar vector for OE‐Itga8. These constructs were transformed into bacteria, and positive clones were confirmed by sequencing. The recombinant vectors, along with packaging plasmids, were co‐transfected into HEK293T cells. Viral supernatants were collected 48–72 h later, concentrated, and titrated. Transduction was performed at an optimal multiplicity of infection (MOI). The shRNA sequence used in this study is listed in Table S3 (Supporting Information).

### Construction of the Recombinant Rat NPNT Protein

The recombinant rat NPNT protein was constructed by Wuhan AtaGenix Co., Ltd. The rat NPNT protein consists of 606 amino acids, with a C‐Strep tag added to the original Met1‐Cys606 sequence. The gene sequence was cloned into the pATX2 expression vector for mammalian expression. The construct was transformed, and single colonies were selected and cultured. The plasmid was then transfected into XtenCHO cells, and samples were collected for analysis on day 6 post‐transfection. The protein was purified and stored at −80 °C after verification by SDS‐PAGE and western blotting.

### Cell Contraction Assay

Cell contraction assays were performed using Corning rat tail collagen. Sterile solutions of 10× PBS and 1 mol L^−1^ NaOH were prepared. A 4 mL mixture was made by adding 0.4 mL of 10× PBS, 35 µL of 1 mol L^−1^ NaOH, 1.945 mL of ddH_2_O, and 1.52 mL of 3.96 mg mL^−1^ Corning Collagen I on ice. Cell suspensions (1 × 10⁵ cells mL^−1^) of fibroblasts transfected with either an empty vector or sh‐Itga8 lentivirus were added to the mixture. The mixture (600 µL per well) was plated in 24‐well plates, and the gels were allowed to solidify at 37 °C for 30 min. After the gels were detached from the well edges, complete medium was added according to the experimental group. Contraction was assessed by photography, and the contraction area was quantified.

### Cell Scratch Assay

Fibroblasts transfected with the virus were seeded into a 12‐well plate. Once the cells reached confluence, two perpendicular straight lines were scratched in each well using a 200 µL pipette tip. The cells were then continued to be cultured in DMEM medium containing 0.5% FBS for 48 h, with or without the addition of recombinant NPNT (70 nM) or recombinant Fn1 (10 µg mL^−1^) according to the groups. Photographs were taken at 0, 24, and 48 h, and the area of the blank zone was calculated.

### qRT‐PCR

Total RNA was extracted using the FastPure Cell/Tissue Total RNA Isolation Kit (RC112, Vazyme, China) according to the manufacturer's protocol. Tissues were ground into powder under liquid nitrogen, mixed with Buffer RL, and centrifuged to remove genomic DNA (gDNA). RNA was bound to FastPure RNA Columns III, washed with Buffer RW1 and ethanol‐containing Buffer RW2, and eluted with pre‐warmed RNase‐free ddH_2_O. RNA concentration and purity were assessed using a Nanodrop One spectrophotometer. For reverse transcription, the HiScript II Q RT SuperMix kit (R222, Vazyme, China) was used. A reaction mixture was prepared, consisting of the SuperMix, template RNA, and DEPC‐treated water, followed by a two‐step PCR program. Quantitative real‐time PCR (qPCR) was performed using the Taq Pro Universal SYBR qPCR Master Mix (Q712, Vazyme, China), with the reaction system assembled on ice, containing the Master Mix, primers, cDNA template, and DEPC‐treated water. The qPCR program included an initial denaturation, followed by 40 cycles of amplification and a melting curve analysis. Data were analyzed using the 2^−ΔΔCt^ method to calculate the relative expression of target genes, normalized to an endogenous control, enabling comparison of gene expression between experimental groups. The sequences of the probes used in this study are shown in Table S4 (Supporting Information).

### Immunoprecipitation

Immunoprecipitation was performed using the classic Protein A/G Immunoprecipitation Kit (IK‐1004, Biolinkedin, China) following the manufacturer's instructions. Briefly, 500 µL of IP Lysis/Wash Buffer was added to the cells, along with protease inhibitors at a 1:100 dilution, and incubated on ice for 20 min. The lysate was then centrifuged at 12 000 rpm for 10 min at 4 °C, and the supernatant was collected. To capture the protein complexes, the cell lysates were incubated overnight at 4 °C with 10 µg of primary antibody. Following two washes of the Protein A/G magnetic beads, the immunocomplexes were added and incubated at room temperature for 2 h. The magnetic beads were then collected, and the protein complexes were eluted by denaturation using SDS‐PAGE loading buffer for subsequent western blot analysis. This experiment was performed in both primary bladder fibroblasts and 293T cells (ATCC, USA) overexpressing Itga8. To investigate the exogenous binding interactions, the Fn1 recombinant protein (10 µg mL^−1^) was added to the supernatants.

### Plasmid Construction and Cell Transfection

The kinase‐dead FAK mutant (K454R) plasmid was synthesized by and purchased from WZ Biosciences Inc. (Shandong, China). Primary bladder fibroblasts were transfected with the FAK K454R plasmid or the corresponding empty vector using Lipofectamine 3000 according to the manufacturer's protocol. Cells were harvested for functional assays or Western blot analysis 72 h post‐transfection.

### BMDM Isolation and Co‐Culture

SD rat femurs were harvested, and bone marrow cells were isolated by crushing and filtering. Red blood cells were lysed using lysis buffer, and the remaining cells were cultured in high‐glucose DMEM supplemented with 10% FBS and recombinant rat M‐CSF (20 µg mL^−1^; 556902, Biolegend, USA). After 4 days, half of the medium was replaced, and the cells were further cultured for an additional 2 days before induction with complete high‐glucose DMEM for 48 h. Based on the experimental design, IL‐17a (HY‐P78556, MCE, Princeton, NJ, USA) or Trem2 overexpression plasmids were introduced to the cultures, and the conditioned medium was collected to treat the fibroblasts for subsequent experiments.

### Flow Cytometry

Cells were digested with Accutase solution (A6964, Sigma, USA), neutralized with culture medium, and centrifuged at 300 g for 7 min to remove the supernatant. The cells were then washed twice with PBS. For Fc receptor blocking, the pellet was resuspended in 100 µL PBS, and 2 µL of Fc blocking reagent (550270, BD Pharmingen, USA) was added, followed by incubation at 4 °C in the dark for 15 min. After adding 1 mL PBS and centrifuging at 250 g for 5 min to remove the supernatant, surface staining was performed with prepared solutions of anti‐Cd11b (201819, BioLegends, USA) and anti‐Trem2 (824805, BioLegends, USA) antibodies at recommended concentrations, incubated at 4 °C in the dark for 30–40 min. The cells were then washed twice with 1 mL PBS and centrifuged at 250 g for 5 min, inverting the tubes to discard the supernatant. Finally, the cells were analyzed using a flow cytometer (NovoCyte Advantron, Agilent, USA).

Mouse bladders were dissected and minced using surgical scissors. The minced tissue was incubated in 1 mL of PBS containing 0.34 U mL^−1^ Liberase TM (Roche) and 100 µg mL^−1^ DNase at 37 °C for 45–60 min, with gentle shaking every 15 min to ensure thorough digestion. When the tissue achieved a translucent, glass‐like appearance and appeared almost fully digested, digestion was stopped by adding 1 mL of PBS supplemented with 2% FBS and 0.2 µM EDTA. The entire bladder digestion mixture was passed through a 70 µm cell filter to obtain a single‐cell suspension, applying gentle pressure to the remaining tissue on the filter. The cell suspension was resuspended in 100 µL PBS. After adding 2 µL of Fc receptor blocking reagent, the mixture was incubated on ice, protected from light, for 15 min. The cells were then washed with 1 mL PBS and centrifuged at 250 g for 5 min to remove the supernatant. Surface staining was performed using primary antibodies against CD45 (103115, BioLegend, USA), EpCAM (118205, BioLegend, USA), CD31 (102409, BioLegend, USA), and Itga8 (AF4076, R&D, USA) at the recommended concentrations. Subsequently, cells were incubated with Alexa Fluor 488‐conjugated donkey anti‐goat IgG (GB25404, Servicebio, USA) for 30 min at 4 °C, followed by two PBS washes. Flow cytometry analysis was conducted using a NovoCyte Advantron flow cytometer (Agilent, USA), and data were analyzed using FlowJo software. Details of the antibodies used in this study are listed in Table S2 (Supporting Information).

### ELISA

The concentration of Fn1 in the supernatant of macrophage culture medium was determined using an ELISA kit (EK0350, Boster, Wuhan, China). The ELISA procedure involved pipetting standards and samples into a pre‐coated microplate and incubating at 37 °C. Following incubation, biotinylated antibody and ABC solution were added sequentially, with washes using 1× Wash Buffer after each incubation step. Color development was initiated by adding TMB substrate, and the reaction was stopped with Stop Solution. The absorbance was then measured at 450 nm using a plate reader (BioTek Synergy H1, Agilent, USA).

### Virus Injection into the Bladder In Vivo

Virus injection into the bladder wall was performed one week prior to NB modeling, as described by Yuan et al.^[^ ^68^ ^]^ Briefly, a midline laparotomy was performed to expose the bladder, and urine was evacuated. Four injection sites (at the 3, 6, 9, and 12 o'clock positions) on the bladder wall were targeted, with 1 × 10^6^ IU of virus delivered at each site using a 30‐gauge Hamilton syringe (10 µL per site). After the injection, the wound was closed, and the animal was placed on a thermal blanket for recovery.

### Macrophage Depletion

C57BL/6 mice received intraperitoneal injections of clodronate liposomes (CLO lip, 200 µL per dose) or control liposomes (Ctrl lip) three days prior to BPNI. Bladder function was assessed via urodynamics at day 28 post‐BPNI. Histological evaluation was performed using Masson's trichrome staining, and collagen content was quantified by hydroxyproline assay. Immunofluorescence was conducted on the bladder to assess macrophage infiltration and collagen deposition. Quantification was performed on at least three fields per sample using ImageJ.

### Validation of Cre‐Mediated Recombination Specificity

*Col1a2*‐CreERT mice were crossed with *Rosa26*‐tdTomato reporter mice. Tamoxifen (75 mg kg^−1^) was administered intraperitoneally for 5 consecutive days to induce recombination. After a 7‑day washout period, bladder tissues were collected, and frozen sections were immunostained. The percentage of tdTomato‑positive cells co‑localizing with each marker was quantified with ImageJ software.

### Statistical Analysis

scRNA‐seq data were processed and normalized using Seurat's default LogNormalize algorithm. Bulk transcriptome sequencing data were normalized using the FPKM method. Details are provided in the corresponding Methods section. All quantitative data are presented as means ± standard deviations (SD). The sample size (n) for each experiment is indicated in the figure legends. For normally distributed data, two‐group comparisons used unpaired two‐tailed Student's *t*‐tests; multiple group comparisons used one‐way ANOVA followed by Tukey's post‐hoc test; and comparisons across multiple time points between two groups used two‐way ANOVA. The significance threshold was *p* < 0.05. Asterisks or pound signs denote significance levels as follows: */# *p* < 0.05; **/## *p* < 0.01; ***/### *p* < 0.001. Statistical analysis was performed using GraphPad Prism 8. Urodynamic data from the BL‐420N system were analyzed with Origin 2022.

## Conflict of Interest

The authors declare no conflict of interest.

## Author Contributions

J.W. and S.W. contributed equally to this work. Q.L., J.L. conceived the project and supervised the study; Q.L. guided the study design and data analysis. L.R., X.L., W.X. and S.Z. designed the experiments, performed the research, analyzed the data and prepared the figures; L.Z. and P.H. performed part of experiments or provided technical assistance; J.W. and S.W. wrote or refined the manuscript. All authors approved the final version of the manuscript.

## Supporting information



Supporting Information

## Data Availability

The scRNA‐seq data supporting this study are available in the GEO repository under accession number GSE305964. Other data supporting the findings of this study are available from the corresponding author upon reasonable request.
