# Supplementary material for: Targeting Itga8 Mitigates Neurogenic Bladder Fibrosis Driven by Trem2⁺ Macrophage‐Derived Fn1 via FAK/RhoA/ROCK Signaling
Source: Adv Sci (Weinh). 2025 Dec 8;13(9):e10631. doi: 10.1002/advs.202510631 (PMC12904034; doi:10.1002/advs.202510631)
Supplement: Supplementary file 1 — Supporting Information [file ADVS-13-e10631-s001.pdf]

Supporting Information

Title

Targeting Itga8 Mitigates Neurogenic Bladder Fibrosis Driven by Trem2^{+} Macrophage-Derived Fn1 via FAK/RhoA/ROCK Signaling

Jiaxin Wang, Siyuan Wang, Lida Ren, Xinqi Liu, Lei Zhang, Peng Hu, Wenchao Xu, Shuaixiang Zheng, Jihong Liu, Qing Ling**

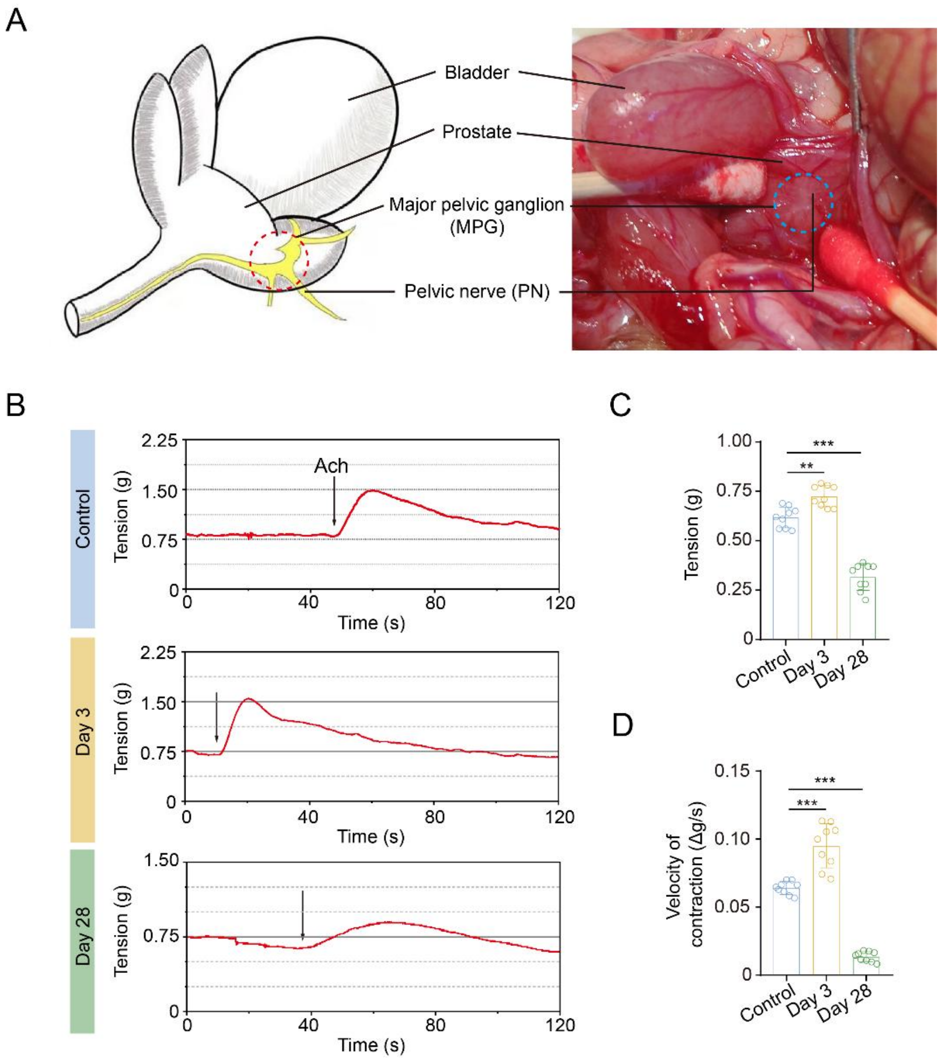

Fig. S1. Characterization of the neurogenic bladder model induced by bilateral pelvic nerve injury. (A) Schematic diagram and representative images of the pelvic ganglion and pelvic nerves, showing the pelvic ganglion located in the posterior-lateral lobe of the prostate (indicated by the red and blue dashed circles), with the pelvic nerves positioned laterally to the pelvic ganglion. **(B)** Representative images of the bladder detrusor muscle strip assay, with black arrows indicating muscle contraction induced by the addition of acetylcholine to the buffer solution. **(C-D)** Quantitative analysis of (C) muscle contraction intensity and (D) contraction velocity in B, n = 9 per group. Ach = acetylcholine. Data represent mean ± SD. One-way ANOVA was used for C and D. ** $P < 0.01$, *** $P < 0.001$.

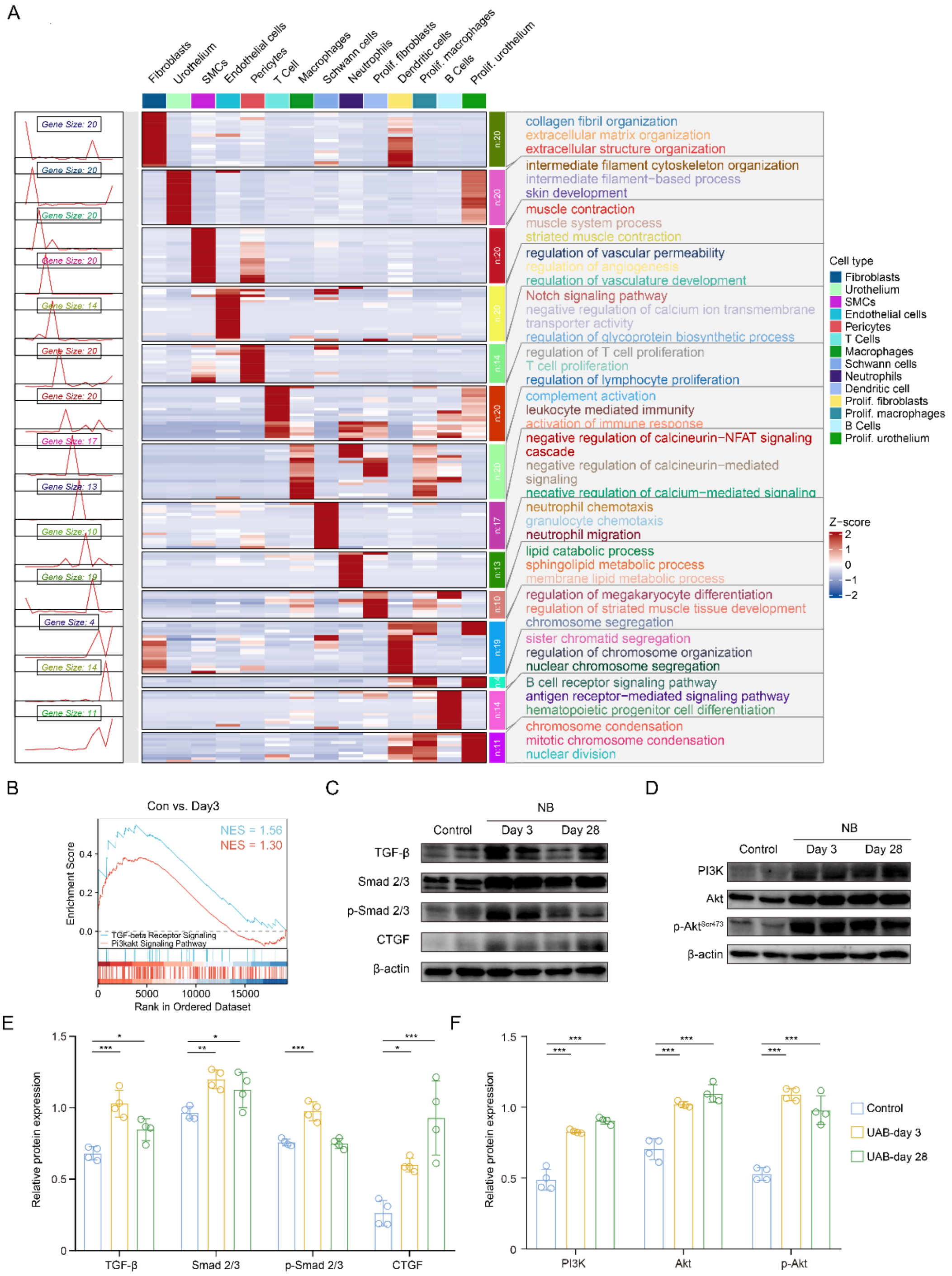

Fig. S2. Single-cell RNA sequencing and RNA-seq reveal that fibrotic pathways are activated early during nerve injury. (A) Representative Gene Ontology (GO) and Kyoto Encyclopedia of Genes and Genomes (KEGG) enrichment of the marker genes expressed in each subset. **(B)** Gene set enrichment analysis (GSEA) revealed upregulation of TGF-β/Smad and PI3K-Akt signaling pathway in the day 3 group. **(C-D)** Representative Western blot images confirming the activation of the (C) TGF-β/Smad and (D) PI3K-Akt signaling pathways during the acute phase of nerve injury. **(E)** Statistical analysis of protein expression in (C), n = 4 per group. **(F)** Statistical analysis of protein expression in (D), n = 4 per group. Data represent mean ± SD. One-way ANOVA was used for C and D. * $P < 0.05$, ** $P < 0.01$, *** $P < 0.001$.

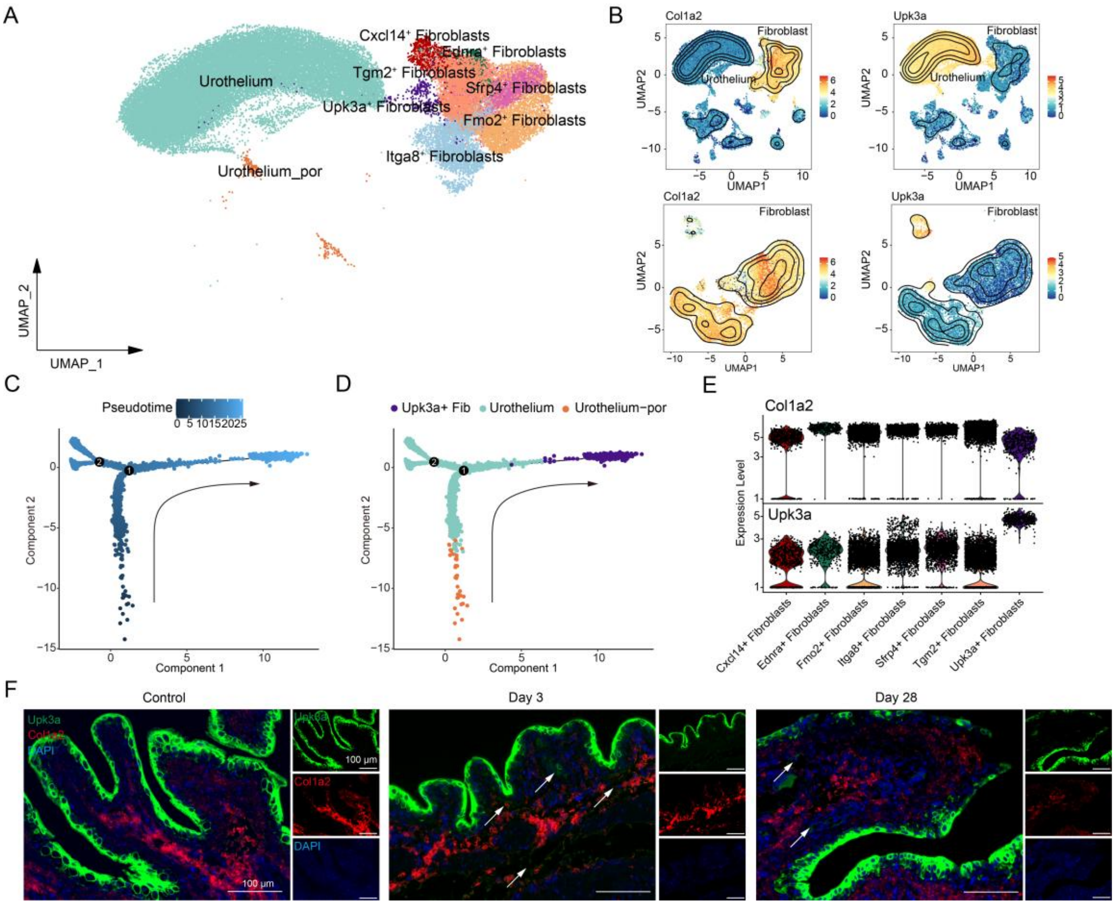

Fig. S3. Transcriptomic and histologic evidence of epithelial-mesenchymal transition in the neurogenic bladder model. (A) UMAP for urothelium and fibroblasts. **(B)** UMAP for the expression of *Col1a2* and *Upk3a* in major cell types and fibroblasts**. (C-D)** Pseudotime analysis of urothelium and Upk3a^{+} fibroblasts. **(E)** Violin plots for the expression of *Col1a2* and *Upk3a* across fibroblast subpopulations. **(F)** Representative immunofluorescence images of Upk3a and Col1a2 in bladder tissues from each group. White arrows indicate Upk3a expression in the submucosal layer.

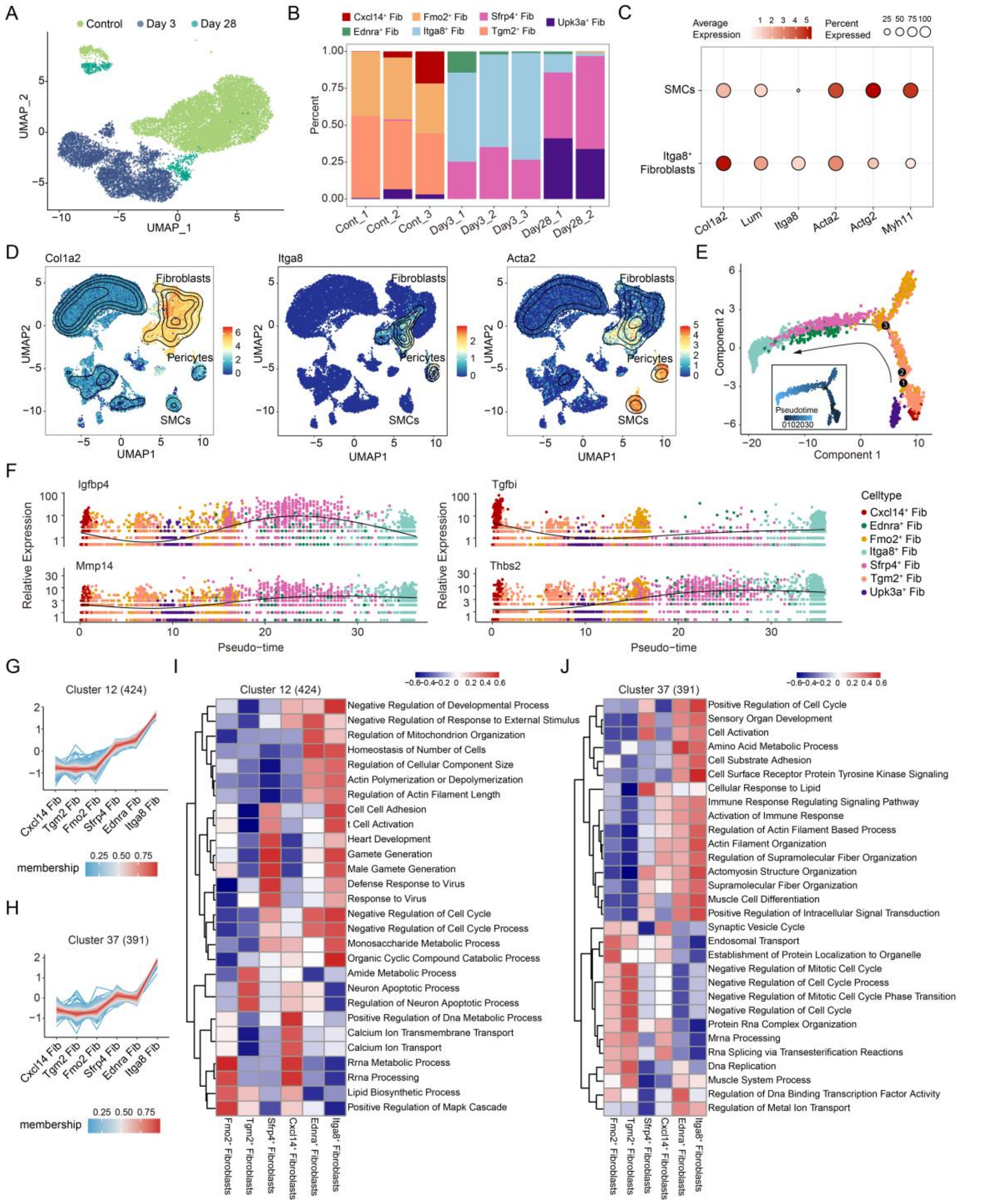

Fig. S4. Single-cell sequencing reveals that Itga8⁺ fibroblasts are a key cell subset involved in bladder fibrosis. (A) UMAP plot showing the distribution of fibroblasts across different groups. **(B)** Statistical analysis of the proportion of fibroblast subpopulations in each sample. **(C)** Dot plot of markers for SMCs and Itga8$^+$ fibroblasts. **(D)** UMAP shows the expression of *Col1a2*, *Itga8* and *Acta2* in major cell types. **(E)** Distribution of different cell types and pseudo-time (in the black box) in fibroblast subtypes. **(F)** Dot plots demonstrated the expression dynamics of *Igfbp4*, *Mmp14*, *Tgfbi*, and *Thbs2* along pseudo-time. **(G-H)** Module-level trajectory analysis performed using ClusterGVis, with all genes divided into 50 modules. Line plots show the significantly upregulated genes in (G) Cluster 12 and (H) Cluster 37 of Itga8$^+$ fibroblasts. **(I)** Enrichment analysis of Cluster 12. **(J)** Enrichment analysis of Cluster 37.

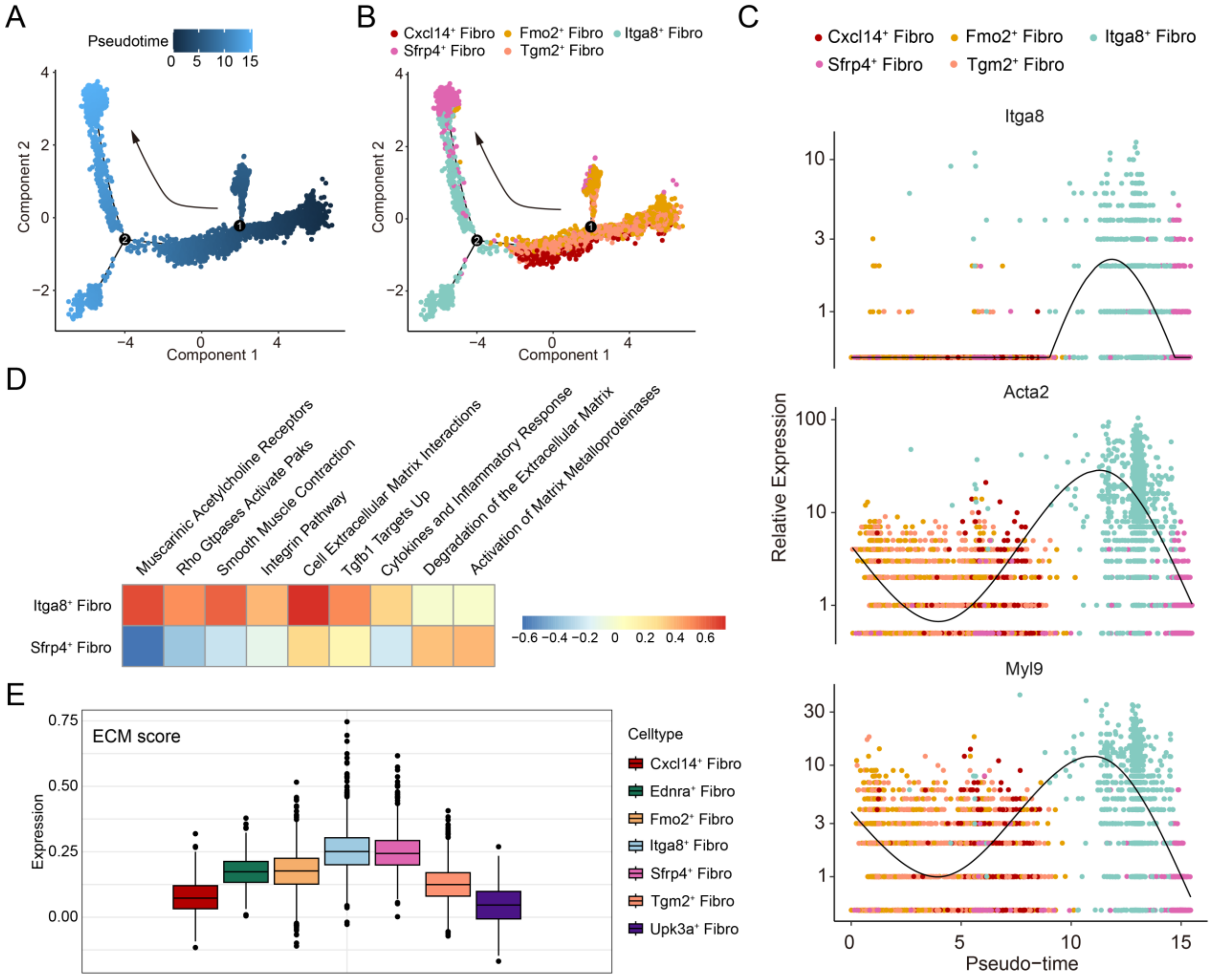

Fig. S5 Comparative analysis identifies Itga8⁺ fibroblasts as an early-appearing and functionally distinct profibrotic subpopulation. (A-B) Pseudotime analysis of fibroblasts. **(C)** Dot plots demonstrate the expression dynamics of *Itga8*, *Acta2*, *Myl9* along pseudo-time. **(D)** Heatmap shows GO/KEGG enrichment of Sfrp4^{+} and Itga8^{+} fibroblasts. **(E)** Box plots of the ECM score for each fibroblast subset.

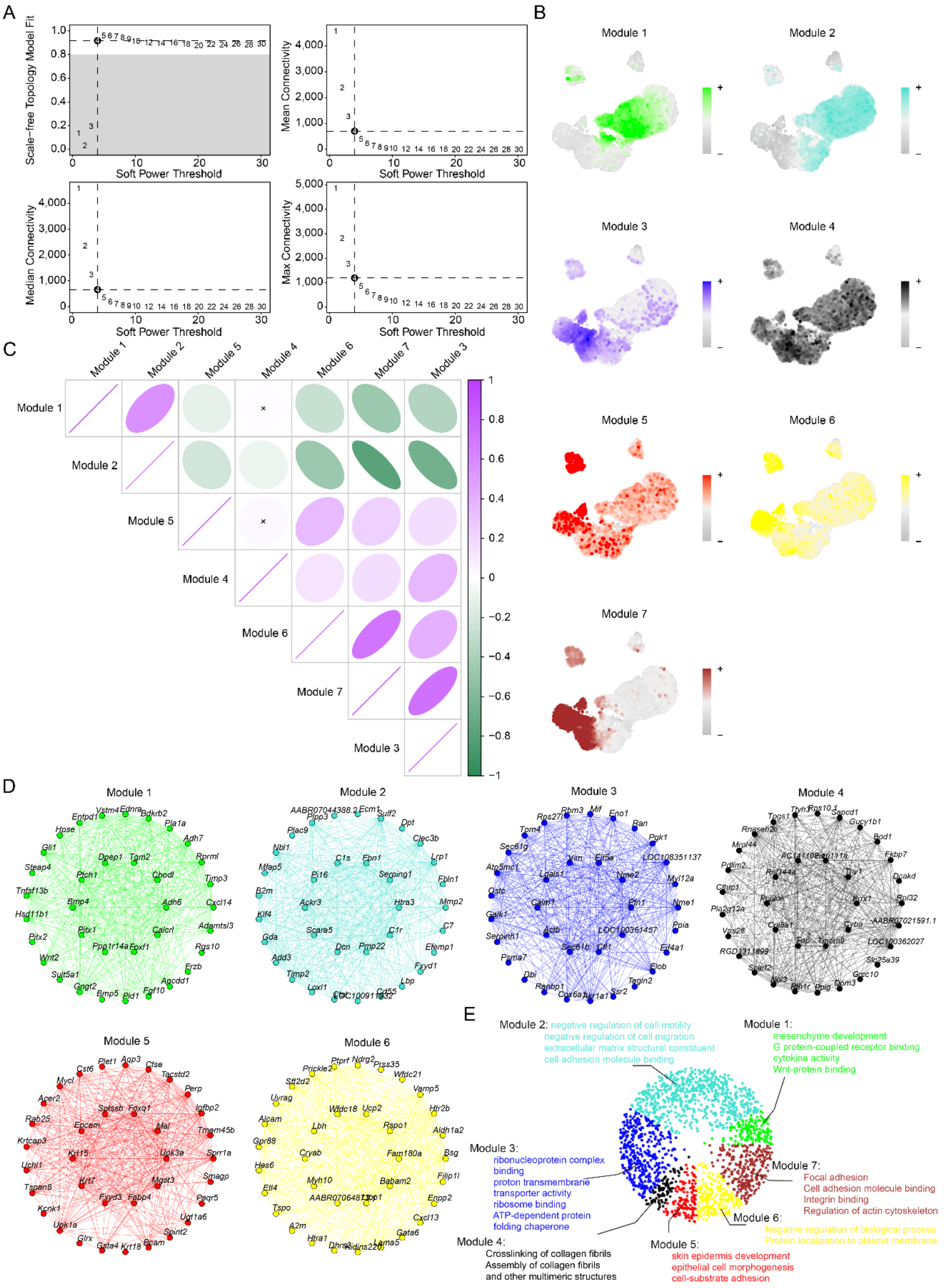

Fig. S6. Identification of fibroblast gene expression modules based on hdWGCNA. (A) Selection of the optimal soft power threshold and the mean, median, and maximum connectivity of the topological network at various minimum soft thresholds. **(B)** UMAP plots showing the expression density of the seven modules. **(C)** Correlation analysis between different modules. **(D)** Networks of the representative genes from module 1-6. **(E)** Enrichment analysis of each module.

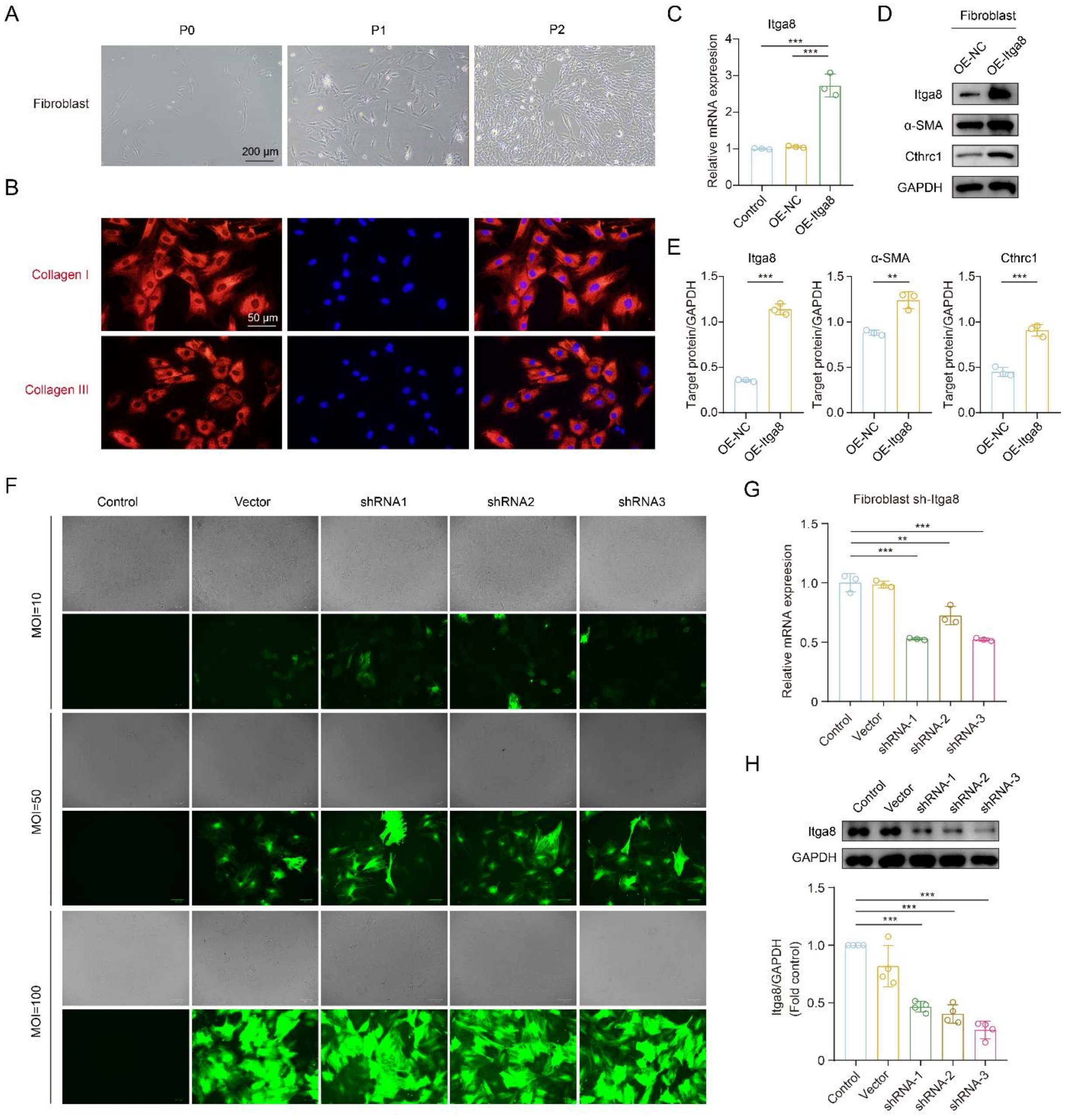

Fig. S7. Isolation, identification, and viral transfection of primary rat bladder fibroblasts. (A) Morphology of rat primary fibroblasts from passage 0 (P0) to passage 2 (P2); **(B)** Representative immunofluorescence results for Collagen I and Collagen III in rat bladder primary fibroblasts at P4. **(C)** qRT-PCR analysis of Itga8 mRNA levels in fibroblasts transduced with OE-Itga8 lentivirus. **(D-E)** Representative western blot images (D) and quantitative analysis (E) of fibroblasts transduced with OE-Itga8 lentivirus. **(F)** Gradient multiplicity of infection (MOI) transfection capability of bladder primary fibroblasts. **(G)** qRT-PCR validation of the knockdown efficiency of three sgRNAs. **(H)** Western blotting validation of the knockdown efficiency of three sgRNAs, confirming that shRNA3 has the highest knockdown efficiency. Data represent mean ± SD. Unpaired two-tailed t-test was used for E. One-way ANOVA was used for C, G and H. ** $P < 0.01$, *** $P < 0.001$.

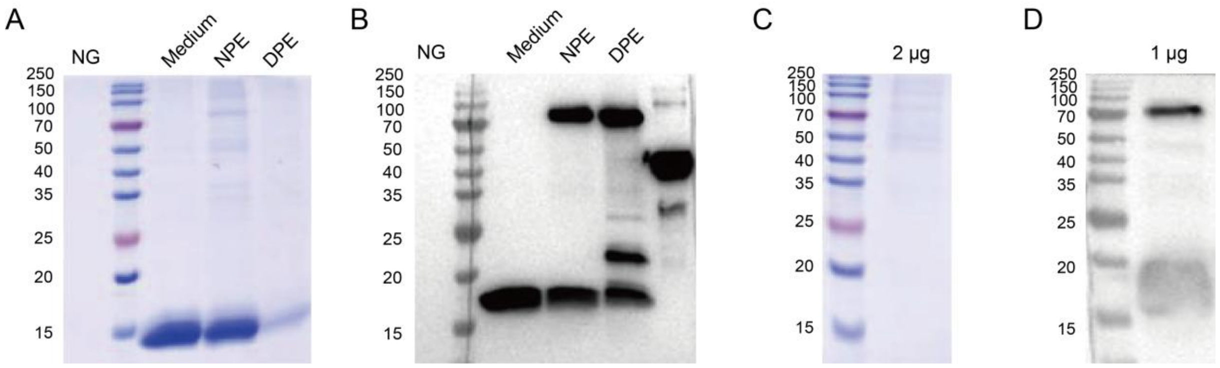

Fig. S8. NPNT Expression and Purification Tests. (A) Expression test with Coomassie Brilliant Blue staining. **(B)** Expression test with anti-Strep-tag antibody western blotting detection. **(C)** Final protein sample with Coomassie Brilliant Blue staining. **(D)** Final protein sample with anti-Strep-tag antibody Western blot detection. NG: Negative control. Medium: Culture supernatant. NPE: Supernatant after cell ultrasonic disruption and centrifugation. DPE: Supernatant after dissolving the pellet with PBS and 8M urea following cell ultrasonic disruption and centrifugation.

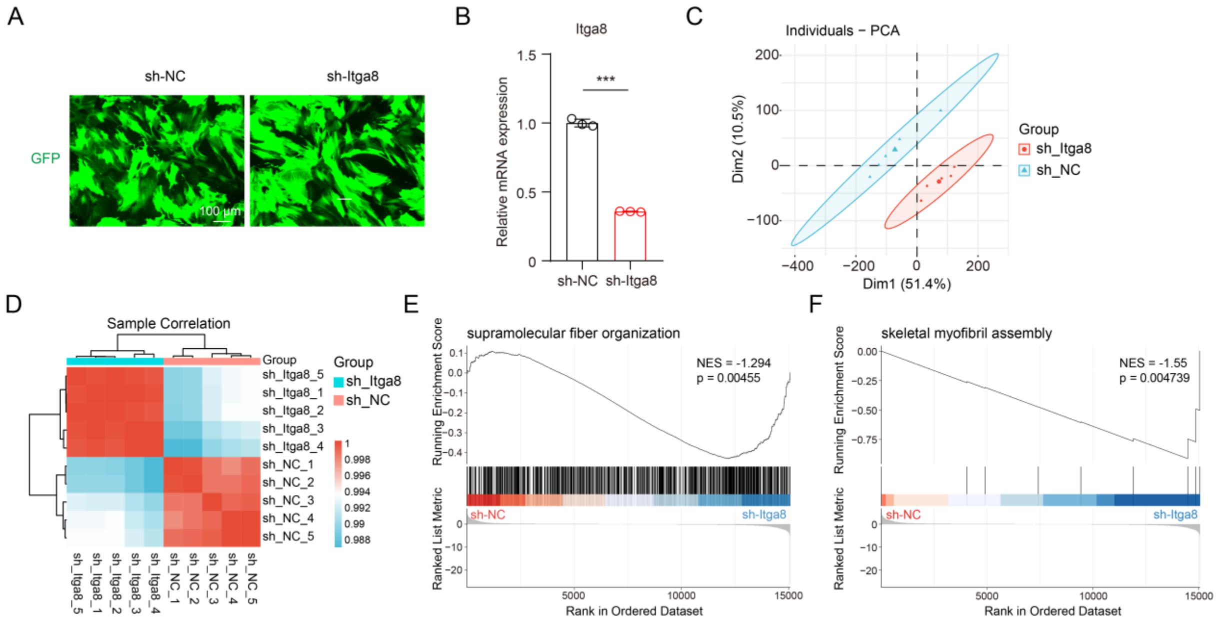

Fig. S9. Transcriptomic profiling of Itga8-silenced fibroblasts. (A) Representative fluorescence microscopy images showing GFP expression in primary bladder fibroblasts following lentiviral transduction. **(B)** Quantitative PCR validation of Itga8 knockdown efficiency. **(C)** Principal component analysis (PCA) plot of the RNA-seq data demonstrates clear separation between control (sh_NC) and Itga8 knockdown (sh_Itga8) groups. Each point represents an independent biological replicate (n = 5 per group). **(D)** Sample correlation heatmap of the RNA-seq data. **(E)** Gene set enrichment analysis (GSEA) revealed downregulation of the supramolecular fiber organization pathway in the sh-Itga8 group. **(F)** GSEA revealed downregulation of the skeletal myofibril assembly pathway in the sh-Itga8 group. Unpaired two-tailed Student's t-test was used for B. $^{***}P < 0.001$

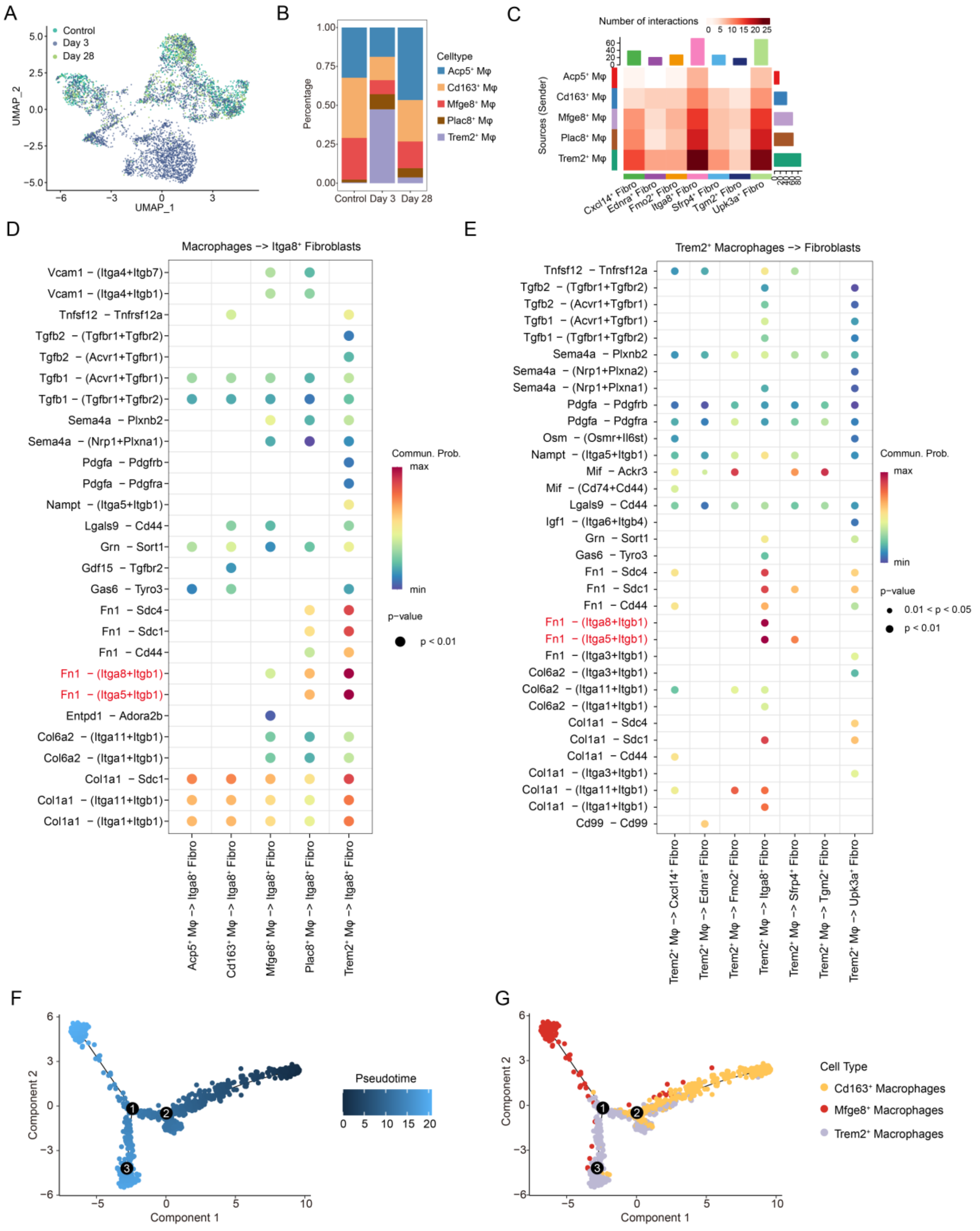

Fig. S10. Single-cell sequencing reveals a significant increase in Trem2⁺ macrophages during the acute phase of nerve injury. (A) UMAP plot showing the distribution of macrophages across different groups. **(B)** Frequency of each cluster on different time points. **(C)** Heatmap of the number of interactions between macrophages and fibroblasts. **(D)** Bubble plot showing the ligand-receptor pairs involved in the interaction between macrophages and Itga8⁺ fibroblasts. **(E)** Bubble plot showing the ligand-receptor pairs involved in the interaction between Trem2⁺ macrophages and fibroblasts. **(F)** pseudo-time in macrophage subtypes. **(G)** Distribution of different cell types in macrophage subtypes.

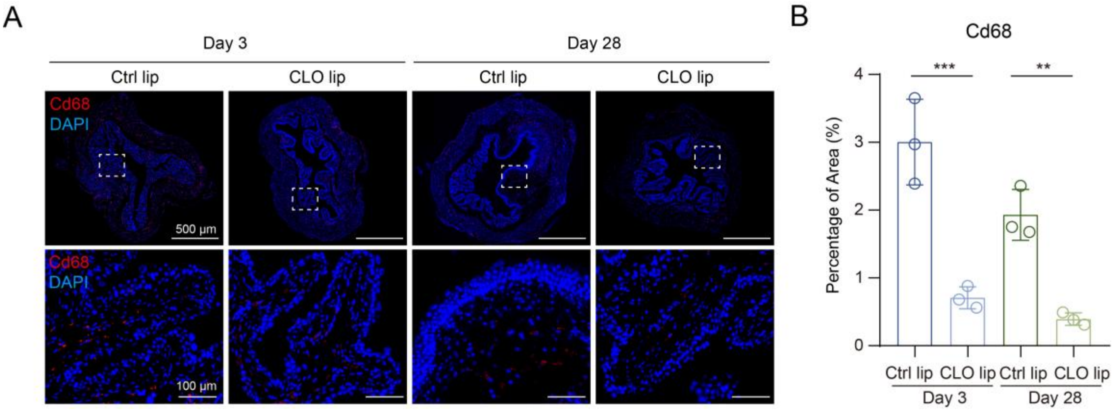

Fig. S11. Validation of macrophage depletion efficiency by clodronate liposomes. (A) Representative immunofluorescence images of Cd68 in bladder sections from Ctrl lip and CLO lip groups at 3 and 28 days post-BPNI. **(B)** Quantitative analysis of Cd68 in immunofluorescence staining. One-way ANOVA was used for B. $^{**}P < 0.01$, $^{***}P < 0.001$.

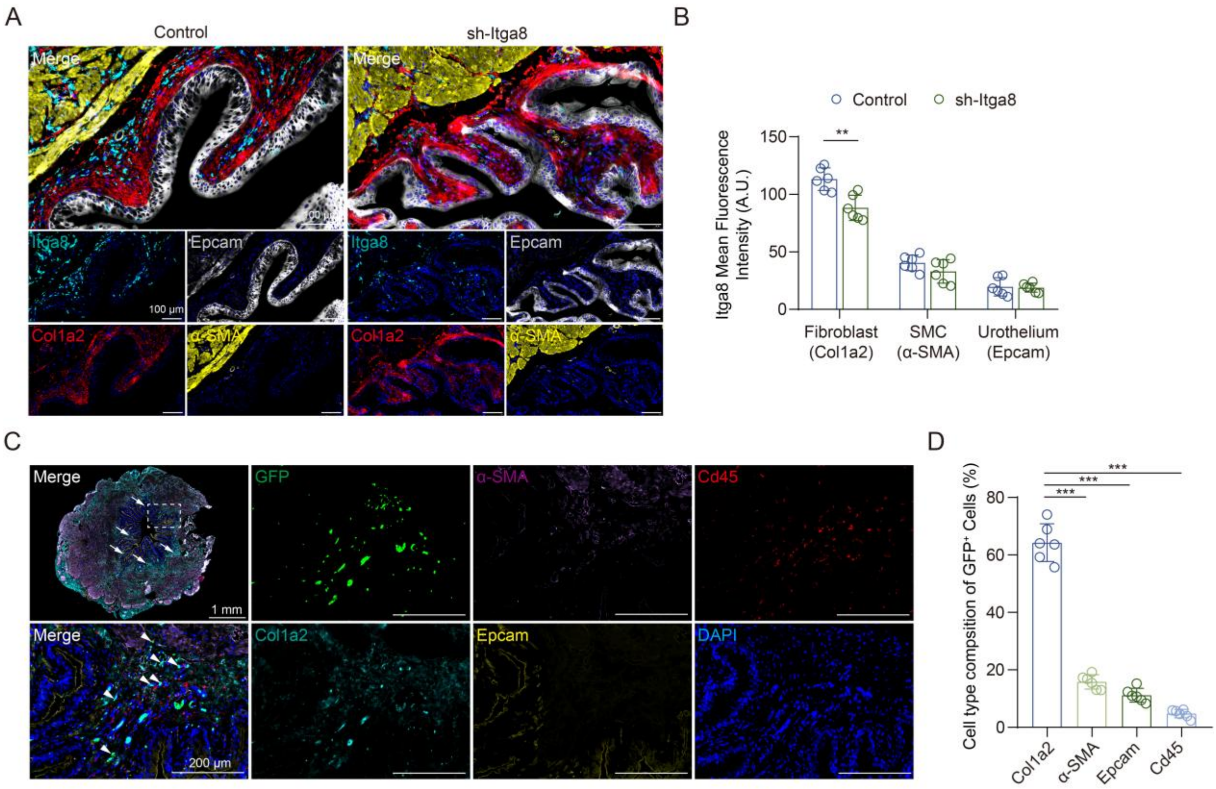

Fig. S12. Validation of fibroblast-enriched Itga8 expression and lentiviral transduction specificity. (A) Representative multiplex immunohistochemistry images showing Itga8, Col1a1, α-SMA and Epcam in the bladder tissues **(B)** Itga8 mean fluorescence intensity of fibroblast, smooth muscle cell (SMC) and urothelium in control and sh-Itga8 group, n = 6 per group. **(C)** Immunofluorescence analysis of bladder after GFP-expressing lentivirus delivery, visualizing transduced cells (green) in relation to specific cellular markers. White arrow: GFP^+ cells in the submucosal layer. White triangle: fibroblasts co-localizing GFP and Col1a2. **(D)** Cell type composition of GFP^+ cells across fibroblasts (Col1a2), SMC (α-SMA), urothelial cells (Epcam) and immune cells (Cd45), n = 6 per group. Unpaired two-tailed Student's t-test was used for B. One-way ANOVA was used for D. $^{**}P < 0.01$, $^{***}P < 0.001$.

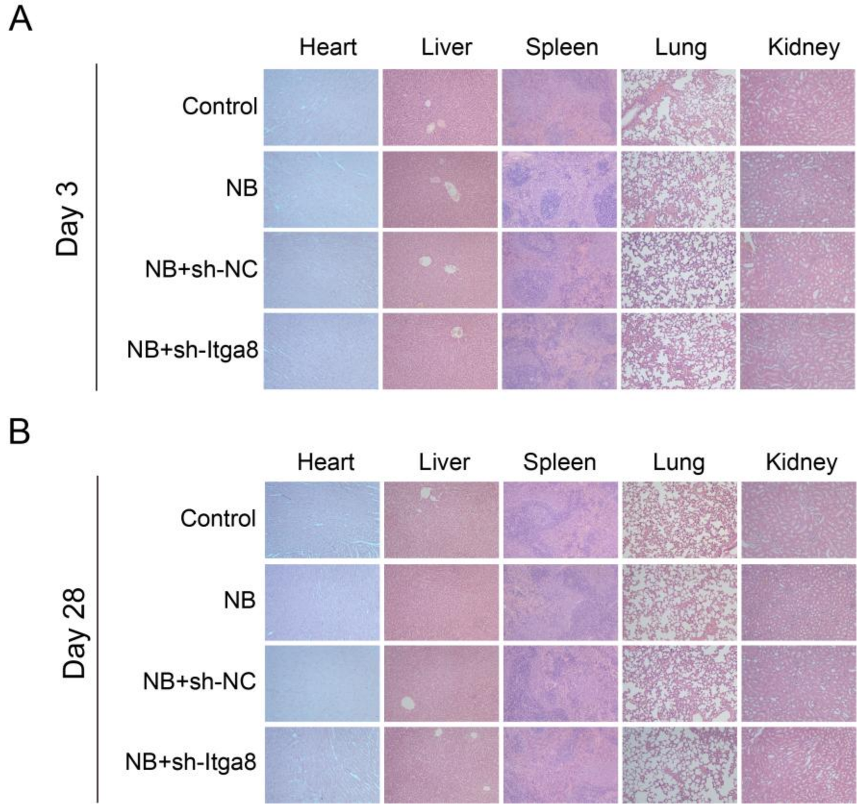

Fig. S13. Intraperitoneal injection of sh-Itga8 has no significant effect on major organs. **(A)** Representative HE staining images of heart, liver, spleen, lung, and kidney during the acute phase of each group of BPNI. **(B)** Representative HE staining images of heart, liver, spleen, lung, and kidney during the chronic phase of each group of BPNI.

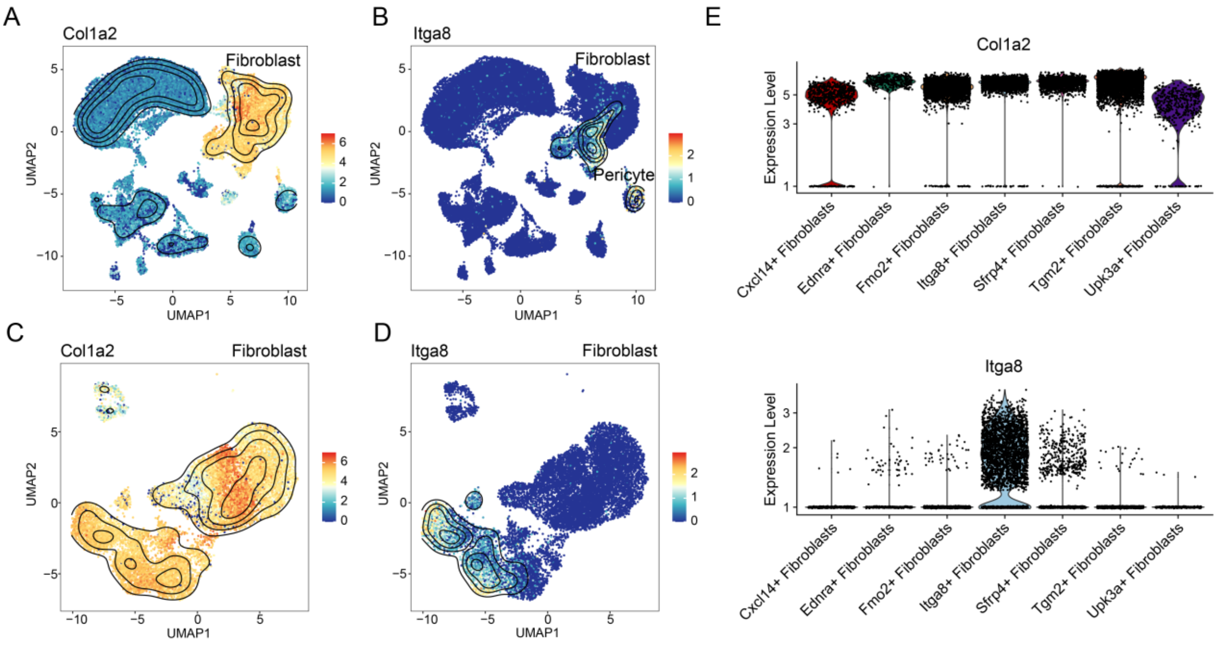

Fig. S14. Single-cell transcriptomic evidence confirms fibroblast-specific co-expression of *Col1a2* and *Itga8*. (A) UMAP shows the expression of *Col1a2* in major cell types. **(B)** UMAP shows the expression of *Itga8* in major cell types. **(C)** UMAP shows the expression of *Col1a2* in fibroblasts. **(D)** UMAP shows the expression of *Itga8* in fibroblasts. **(E)** Violin plots depict the expression of *Col1a2* and *Itga8* across fibroblast subpopulations.

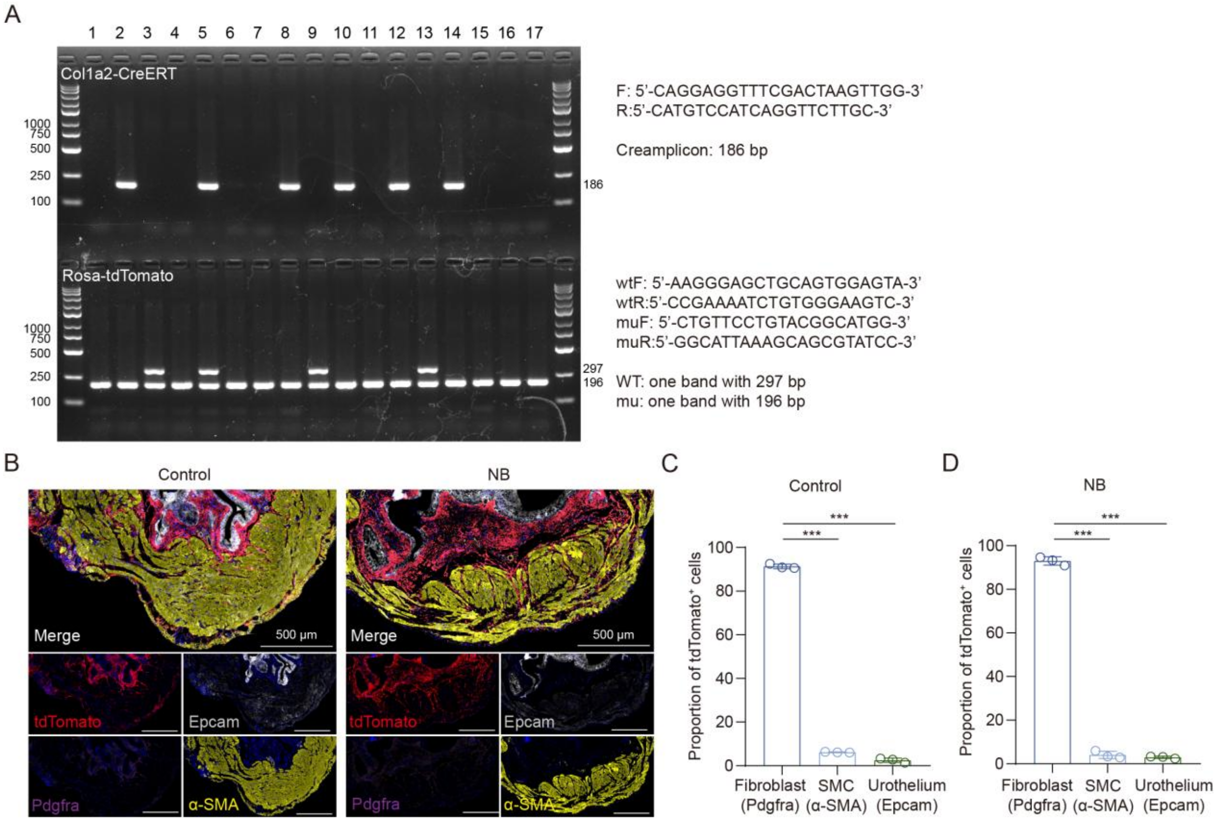

Fig. S15. Specificity of *Col1a2*-CreERT-mediated recombination in bladder tissues. (A) Representative image of genotyping PCR of the *Col1a2*-CreERT and *Rosa26*-tdTomato alleles. **(B)** Representative immunofluorescence images of bladder sections from *Col1a2*-CreERT; *Rosa26*-tdTomato mice in control and NB group. **(C)** Quantification of the proportion of $tdTomato^{+}$ cells in control group, n = 3 per group. **(D)** Quantification of the proportion of $tdTomato^{+}$ cells in NB group, n = 3 per group. One-way ANOVA was used for C and D. $^{***}P < 0.001$.

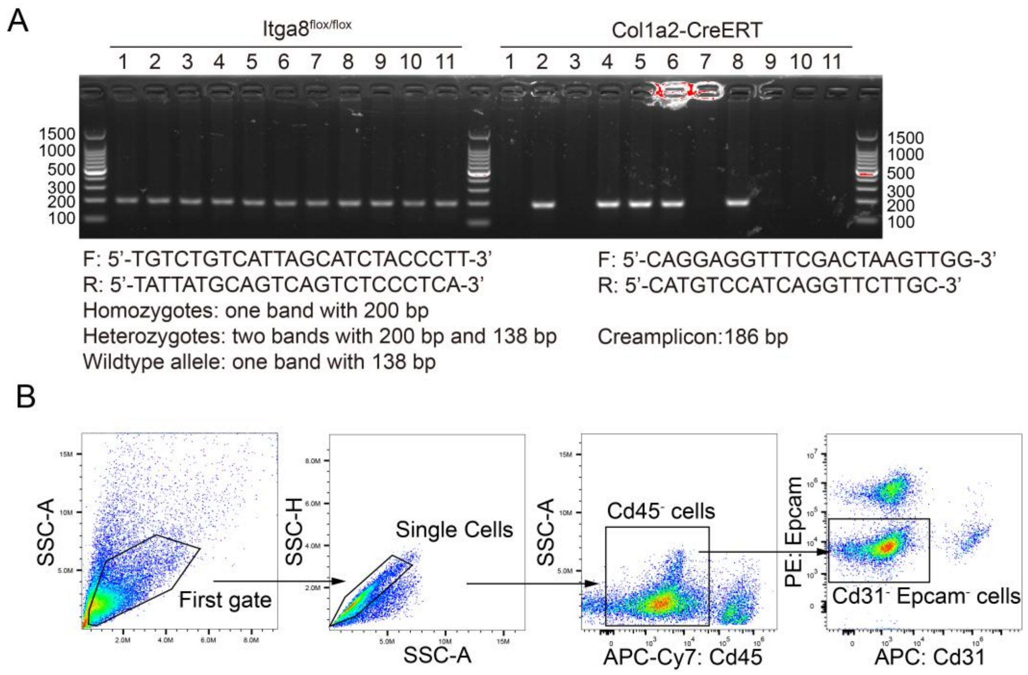

Fig. S16. Identification of *Col1a2*-CreERT; *Itga8*$^{fl/fl}$ conditional knockout mice. (A) Representative images show that all mice are *Itga8*$^{fl/fl}$ homozygotes, with the right panel indicating CreERT$^+$ mice by the band at 186 bp. **(B)** Flow cytometry gating strategy for the identification of fibroblasts in bladder tissue.

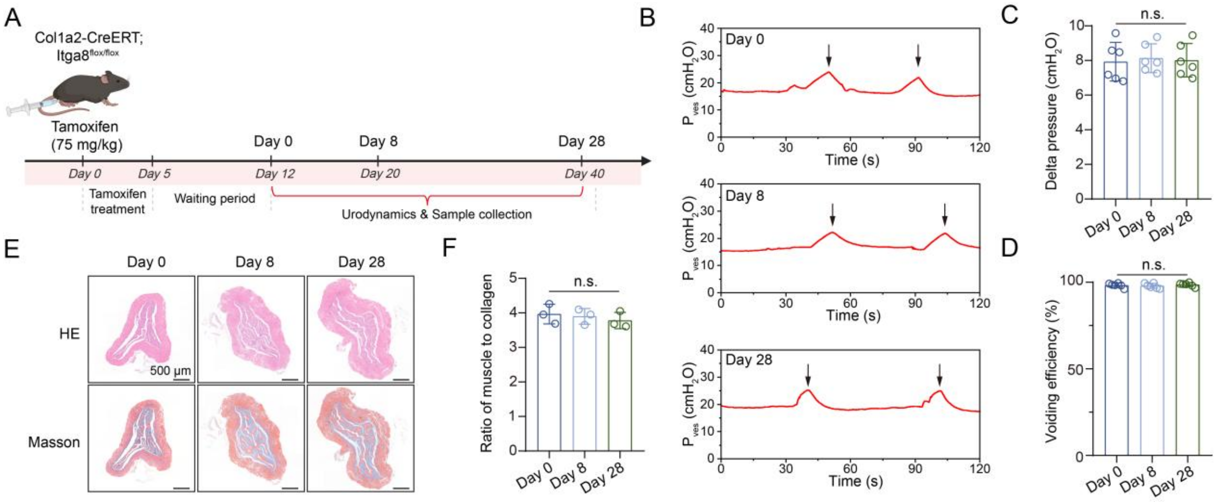

Fig. S17. Fibroblast-specific deletion of Itga8 does not impair normal bladder structure or function. (A) Schematic diagram of the animal experimental design. **(B)** Representative urodynamic profiles in *Col1a2*-CreERT^{+}; *Itga8*$^{fl/fl}$ mice, and the black arrow indicates a single voiding event. **(C-D)** Bar graphs representing (C) delta pressure and (D) voiding efficiency (calculated as voided volume/maximum bladder capacity) for each group, n = 6 per group. **(E)** Representative images of Hematoxylin-eosin staining and Masson's trichrome staining for each group. **(F)** Quantification of ratio of smooth muscle to collagen in (E), n = 3 per group. One-way ANOVA was used for C, D, F. n.s. = not statistically significant.

Table S1. Gene set scoring

Gene set	Genes
ECM score	"Itga2","Col5a1","Lamc3","Dcn","Lama3","Cpb2","Itga11","Prss2","Cyp1b1","Col6a3","Col6a2","Col6a1","Col11a1","Fscn1","Ctrb2","Hspg2","Fap","Plod3","Grem1","Col4a1","Col3a1","Col2a1","Col1a1","Fermt1","Nphs1","Lama2","Thbs1","Lamb1","Vcam1","Bcan","Ctrb1","Tnxa","Abl1","Bcl3","Dpp4","Prdx4","Olfml2a","Sparc","Fn1","Ccdc80","Mmp11","Loxl1","Ddr1","Capn2","Tll1","Capn1","Mpv17","Anxa2","Pdgfa","Cma1","Vtn","Ctsv","Matn3","Lama5","Itga10","Col8a2","Col18a1","Adamts20","Adam19","Lama4","Reck","Col5a2","Mmp20","Spink5","Ric8a","Ddr2","Spint2","Comp","Ctgf","Clasp2","Serac1","Flot1","Carmil2","Icam5","Eng","Atxn1l","Mmp8","Serpine1","Fmod","Nid2","Fgf2","Antxr1","Vwa1","Thsd4","Tnxb","Itgax","Itgal","Spp1","Col19a1","Gfod2","Atp7a","Noxo1","Dnajb6","Adamts4","Adam12","Fgfr4","Matn4","Adamts2","Col5a3","Ihh","Ero1a","Sfrp2","Eln","Ctss","Kdr","Klk2","Smoc2","Ndnf","Fbln5","Vps33b","Optc","Smad3","Spint1","Plg","Prss1","Nr2e1","Creb3l1","Icam4","Itgb1","Mmp14","Lrp1","P4ha1","Col7a1","Itga4","Vcan","Adam10","Abi3bp","Dpt","Meltf","Dspp","Cd47","Nox1","Col8a1","Hsd17b12","Ptk2","Crispld2","Pdgfb","Lox","Has2","Mmp19","Col1a2","Spock2","Col16a1","Itga8","Ibsp","Bgn","Scube3","Tcf15","Serpinh1","Ttr","Cd44","Jam2","Lcp1","Vipas39","Cflar","Gas6","Itgb2","Mmp1","Klkb1","App","Foxc1","B4galt1","Myh11","Phldb2","Lamb2","Nfkb2","Ctsg","Acan","Itgb6","Lamb3","Itga9","Tgfb2","Elane","Nphp3","Clasp1","Mfap2","Fbln1","Kif9","Pecam1","Matn1","Itgam","Hpn","Vwf","Mmp12","Col12a1","Foxc2","Hpse2","A2m","Tpsab1","F11r","Col13a1","Has1","Ctsk","Itgae","Apbb2","Loxl2","Itgb5","Ercc2","Mpzl3","Sh3pxd2a","Nf1","Col4a4","C6orf15","Col9a1","Cst3","Timp1","Agt","Adamts14","Lamc1","Tgfb1","Pomt1","Itgav","Sox9","Cyr61","Egflam","Tll2","Serpinb5","Adamtsl4","Exoc8","Pdpn","Csgalnact1","Tnfrsf11b","Pdgfra","Htra1","Mmp16","Mmp15","Loxl3","Col10a1","Postn","Ets1","Timp2","Capns1","Scx","Tmprss6","Itga7","Chadl","Notch1","Myf5","Itgb8","Itgb7","Adamtsl2","Itga3","Furin","Wnt3a","Tnc","Kazald1","Lum","Npnt","Vit","Tnf","Col27a1","Washc1","Tgfb1","Agrn","Adam8","Nid1","Sh3pxd2b","Gpm6b","Madcam1","Adam15","Jam3","Tnr","Col14a1","Ero1b","Tgfb1","Dag1","Ramp2","Itgb3","Bsg","Myo1e","Foxf2","Foxf1","Lama1","Col11a2","Col9a2","Egfl6","Lamc2","Phldb1","Itga2b","Elf3","Mmp

	9","Col4a2","Mmp2","Mmp3","Hapln1","Mfap4","Scube1","Ecm2","Sulf1","Sulf2","Has3","Itgb4","Itga6","Pxdn","Mmp13","Icam2","Ncan","Col9a3","Col4a6","Fbn2","Fbn1","Hapln2","Bmp1","Mfap5","Serpinf2","Itga5","Col4a5","Icam3","Ctsl","Flrt2","Klk7","Icam1","Capns2","Adamts5","Rgcc","Itgad","Col4a3","Dmp1","Fgg","Fgb","Fga","Itga1","Adamts3","Cdh1","Wt1","Mmp10","Mmp7"
Collagen score	"Col10a1","Col12a1","Col14a1","Col16a1","Col1a1","Col1a2","Col21a1","Col3a1","Col5a1","Col5a2","Col6a2","Col6a3","Col8a1","Col8a2","Col11a2","Col13a1","Col15a1","Col17a1","Col23a1","Col25a1","Col4a1","Col4a2","Col22a1","Col24a1","Col26a1","Col4a6","Col9a2","Col18a1","Col19a1","Col27a1","Col4a3","Col4a4","Col4a5","Col6a1","Col7a1","Col28a1","Col5a3","Col9a3"
Pro-fibrosis score	"Lipa","Lpl","Fdx1","Spp1","Sparc","Matk","Gpc4","Palld","Chi3l1","Chit1","Ctsk","Mmp9","Mmp7","Csf1","Fcmr","Timp3","Siglec15","Ccl22"
anti-fibrosis score	"Mmp1","Mmp2","Mmp14","Mmp13","Itga2","Mrc1","Mrc2","Mfge8"

Table S2. Antibody used in this study

**Primary Antibodies**			
**Antibody**	**Supplier**	**Catalogue Number**	**Concentration**
TGF-β	Affinity	AF1027	1:1000
Smad 2/3	CST	8685	1:1000
p-Smad 2 (Ser465/467) /3(Ser423/425)	CST	8828	1:1000
CTGF	Abclonal	A11067	1:1000 for WB and 1:200 for IF
PI3K	Proteintech	55036-1-AP	1:1000
Akt	CST	9272	1:1000
p-Akt (Ser473)	Boster	BM4838	1:1000
β-actin	Proteintech	20536-1-AP	1:1000
Krt18	Servicebio	GB15232	1:200
Col1a2	Abclonal	A21059	1:100
Cd68	Servicebio	GB113109	1:200
α-SMA	Servicebio	GB111364	1:200
Cthrc1	Proteintech	16534-1-AP	1:1000 for WB and 1:200 for IF
Itga8	R&D	AF4076	1:1000 for WB and 1:200 for IF
Itga5	Proteintech	10569-1-AP	1:1000
Itgb1	Proteintech	12594-1-AP	1:1000
Fn1	Abclonal	A12932	1:1000
GAPDH	Proteintech	60004-1-Ig	1:5000
p-FAK	Abclonal	AP1447	1:1000
FAK	Abclonal	A11131	1:1000 for WB and 1:200 for IF
RhoA	Proteintech	10749-1-AP	1:1000
ROCK1	Proteintech	21850-1-AP	1:1000
ROCK2	Proteintech	21645-1-AP	1:1000

Myl9	Abclonal	A3039	1:1000	
Col1a1	Abclonal	A16699	1:100	
Col3a1	Servicebio	GB111629	1:100	
Trem2	Abclonal	A10482	1:1000 for WB and 1:200 for IF	
Pdgfra	Abclonal	A26747	1:100	
Upk3a	Abclonal	A28188	1:100	
Cd45	Servicebio	GB113886	1:100	
**Secondary Antibodies**				
**Antibody**	**Supplier**	**Catalogue Number**	**Conjugate**	**Concentration**
Goat anti rabbit	Boster	BM2006	HRP	1:5000
Goat anti mouse	Abcam	ab6789	HRP	1:5000
Donkey anti goat	Servicebio	GB23404	HRP	1:5000
Donkey anti goat	Servicebio	GB25404	488	1:200
Goat anti rabbit	Servicebio	GB21404	Cy3	1:200
Goat anti rabbit	Servicebio	GB25303	488	1:200
Tyramide	Servicebio	G1232	647	1:500
Tyramide	Servicebio	G1250	440	1:500
Tyramide	Servicebio	G1231	488	1:500
Tyramide	Servicebio	G1251	546	1:500
**Flow Cytometry**				
**Antibody**	**Supplier**	**Catalogue Number**	**Concentration**	
Cd11b	Biolegend	201819	1:100	
Trem2	Biolegend	824805	1:100	
CD45	Biolegend	103115	1:100	
EpCAM	Biolegend	118205	1:100	
CD31	Biolegend	102409	1:100	

Table S3. Itga8-shRNA sequence

Primer	Sequence
Negative Control	TTCTCCGAACGTGTCACGT
shRNA1	CGATTAAGACATCGGTTATTT
shRNA2	AGATCAACCCGCAGGATATAA
shRNA3	CCAGATAAGCAGGAGATAATT

Table S4. qRT-PCR sequence

Primer	Sequence
Itga8-F	TACAACGGAAACGCCAGAGG
Itga8-R	CCCCGACAAGTAAATCTGGGT
Acta2-F	CATCCGACCTTGCTAACGGA
Acta2-R	AGTCCAGCACAATACCAGTTGT
Cthrc1-F	AGGGAAGTGGTAGACCTGTATAATG
Cthrc1-R	TGCTTGTAGTTTGGGGTCCA
Fn1-F	ATGAGAAGCCTGGATCCCCT
Fn1-R	CAGTTGGGGAAGCTCATCTGT
Trem2-F	CAACTTCAGATCCTCACTGGACC
Trem2-R	GGTGGAGGGGAGAGTATGCC
β-actin-F	ATCATTGCTCCTCCTGAGCG
β-actin-R	GAAAGGGTGTAAAACGCAGCTC
